# Identification and Experimental Validation of Tumor Antigens and Hypoxia Subtypes of Osteosarcoma for Potential mRNA Vaccine Development

**DOI:** 10.2174/0109298673348041250407062346

**Published:** 2025-04-29

**Authors:** Chunnian Ren, Dawei He, Quan Wang

**Affiliations:** 1 Chongqing Key Laboratory of Pediatrics, Department of Cardiothoracic Surgery, Children’s Hospital of Chongqing Medical University, National Clinical Research Center for Child Health and Disorders, Ministry of Education Key Laboratory of Child Development and Disorders, China International Science and Technology Cooperation Base of Child Development and Critical Disorders, Chongqing, China;; 2 Department of Pediatric Surgery, Chengdu Women's and Children's Central Hospital, School of Medicine, University of Electronic Science and Technology of China, Chengdu, 611731, China;; 3 Department of Urology Surgery, Children’s Hospital of Chongqing Medical University, Chongqing, China

**Keywords:** mRNA vaccine, osteosarcoma, hypoxia subtypes, hypoxia landscape, tumor antigen, hypoxia subtype

## Abstract

**Background:**

Osteosarcoma is the most common primary malignant bone tumor in children and adolescents. The aim of this study was to explore the possibility of OS hypoxia subtype for anti-OS mRNA vaccine development and select suitable patients for precision therapy.

**Methods:**

We comprehensively explored hypoxia-related genes (HRGs) as potential sources of tumor neoantigens in OS patients. Gene set enrichment analysis algorithm and consensus clustering analysis were used to determine immune subtypes and evaluate tumor microenvironment. Estimation of stromal and immune cells in malignant tumour tissues using expression data algorithm was used to assess tumour immune activity. The OS hypoxia landscape was visualized using dimensionality reduction analysis based on the DDRTree algorithm. Assessment of clinical samples and molecular experiments were performed to verify the determined tumor antigens.

**Results:**

Four overexpressed and mutated tumor antigens associated with prognosis and infiltration of antigen-presenting cells were identified and verified by clinical samples and molecular experiments. Furthermore, OS patients were stratified into two OS hypoxia subtypes. Interestingly, patients with the OS hypoxia subtype 1 tumor had a superior survival than those with the OS hypoxia subtype 2 tumor. Distinct expressions of immune checkpoint proteins (ICPs) and immunogenic cell death (ICD) modulators were observed in different immune subtype tumors. Finally, the immune landscape of OS showed a high degree of heterogeneity between individual patients.

**Conclusion:**

This study identified potential antigens for the anti-OS mRNA vaccine as well as different OS hypoxia subtypes, guiding more effective immunotherapeutic strategies and selecting appropriate patients for tumor vaccine therapy.

## 1 INTRODUCTION

OS is the most common primary malignant bone tumor in children and adolescents, which often results in a poor prognosis [1, 2]. The survival rate of patients with metastasis has not significantly improved in the past two decades [2, 3] because OS cells exhibit a high propensity to disseminate and can metastasize to almost any site or organ [4]. Therefore, novel strategies are needed to improve the therapeutic outcomes of OS.

The concept of using mRNA to encode proteins for vaccination received its first *in vivo* validation in 1990 [5, 6]. While conventional vaccine technology relies on the bulk production of a vaccine using mammalian cells, mRNA vaccines turn into the final product only once inside a patient’s cells [6]. Some studies have confirmed that mRNA vaccines can activate specific immune responses and slow down tumor progression [6-8]. Prototypical tumor vaccines have the advantages of relative nontoxicity, minimal non-specific effects, broad therapeutic window, and induction of persistent immunological memory. Therefore, tumor vaccines can overcome drug resistance, adverse reactions, limited therapeutic efficacy, and high costs associated with standard chemotherapy and immunotherapies. However, no mRNA vaccine against OS antigens has been developed thus far, and no subgroup of patients suitable for vaccination has been identified.

Implementing mRNA vaccine technology first relies on selecting stable and highly expressed antigens in tumors, which enable the body to recognize and kill tumors quickly. At the same time, these antigens should significantly affect the immune level of tumors, enabling mRNA vaccine-mediated acquired immune enhancement to be achieved [9]. Moreover, there is great heterogeneity in tumor treatment, and mRNA vaccines cannot be suitable for all types of patients [10]. We need to distinguish better the subgroups that mRNA vaccines are suitable for.

Simultaneously, hypoxia plays an important role in solid tumor development, including OS [9, 10]. The role of hypoxia in the tumor immune response seems to be an attractive and promising therapeutic strategy, especially for drug-resistant malignancies. Recent evidence has revealed a strong association between hypoxia and tumor immune reactions [11]. Hypoxia can reduce the activation level of cytotoxic T lymphocytes in a HIF-1α-dependent manner, resulting in immunosuppression and evasion of immune detection [12]. The above results suggest that hypoxia level is a possible tool for distinguishing the sensitivity of mRNA vaccine therapy.

In this study, we aimed to identify potential tumor antigens for anti-OS mRNA vaccine development. OS hypoxia subtypes for identifying suitable OS patients for vaccination were also investigated. Four overexpressed and mutated tumor antigens associated with prognosis and infiltration of antigen-presenting cells were identified, and OS patients were stratified into two OS hypoxia subtypes (I, II). Our findings might provide valuable information to oncologists and be used for the precise treatment of OS patients and the development of mRNA vaccines.

## 2 MATERIALS AND METHODS

The details of the methods employed are provided in supplementary material. This study was divided into two parts: dry lab (bioinformatics analysis) and wet lab (clinical sample and cell experiments validation) [13]. A brief description of the experimental steps for each part is presented below.

### 2.1 Bioinformatics Analysis

#### 2.1.1 Data Extraction and Pre-processing

The expression profile data of the OS were obtained from the Therapeutically Applicable Research To Generate Effective Treatments (TARGET) database (GDC TARGET-OS dataset) and expression profile datasets GSE21257 [14] and GSE14359 [15] were acquired from Gene Expression Omnibus (GEO) database [16] by using the R package Equerry. The specific dataset information is provided in Table **1**.

HRGs were collected from the GeneCards database [17], Molecular Signatures Database (MSigDB) [18], and the list of HRGs provided in the published literature on the PubMed website [19] with the keyword “hypoxia”. 689 HRGs were obtained (Table **S1**). 25 ICD genes and 46 ICP genes recognized as targets for immune activation and response were obtained from the published literature on the PubMed website [20, 21]. The specific gene names are shown in Table **S2**.

#### 2.1.2 Osteosarcoma-related Overexpressed Genes

In the differential analyses of the expression profile data of the TARGET-OS, GSE21257, and GSE14359 datasets, the genes screened out with the criteria of |logFC|>0.5 and *p*-value < 0.05 were identified as overexpressed genes [22]. The genes obtained from a minimum of two sets of data were identified as overexpressed genes in OS.

#### 2.1.3 Prognostic Survival Analysis of Overexpressed Genes and Antigen-presenting Cell Correlation Analysis

Overexpressed genes according to clinical prognostic information in TARGET-OS dataset samples and GSE21257 datasets were combined. Overexpressed genes with a prognostic survival Kaplan‒Meier (KM) curve and a *p*-value < 0.05 were included in the subsequent study.

Immune infiltration analysis was performed on the TARGET-OS OS tissue FPKM format dataset matrix using the ssGSEA algorithm in the GSEA package [23] to evaluate the infiltration abundance of 28 immune cells in the tumor microenvironment.

#### 2.1.4 Identification and Validation of Hypoxia Subtypes

A univariate Cox regression analysis was performed based on the expression of HRGs in the TARGET-OS dataset and GSE21257 dataset. The consensus clustering method [24, 25] was employed to analyse the HRGs, identifying different OS hypoxia subtypes.

#### 2.1.5 Differential Analysis of ICD and ICP Expression in Different Hypoxia Subtypes and Immune Evaluation

The differences in immune infiltration characteristics of OS patient samples between different OS hypoxia subtypes were evaluated based on the ESTIMATE package. Differential analysis of the expression of ICD and ICP genes in OS patient samples between different OS hypoxia subtypes was performed as well.

#### 2.1.6 Identification of Immune Infiltrating Cells among Different OS Hypoxia Subtypes in OS Datasets

The differences in the infiltration abundance of immune cells between the different OS hypoxia subtypes of OS datasets (TARGET-OS and GSE21257 datasets) were analysed by ssGSEA algorithm [26, 27] and displayed using heatmaps.

#### 2.1.7 Hypoxia Landscape Construction of the Tumor Microenvironment (Pseudotime Analysis)

The distribution in individual patients of the OS hypoxic subtype of the TARGER-OS dataset was analysed by pseudotime analysis model based on the inverse graph embedding (DDRTree) algorithm [28]. Finally, color-coded functional maps of hypoxic subtypes of OS cell trajectories were used to visualize the hypoxic landscape.

#### 2.1.8 Weighted Gene Coexpression Network Analysis (WGCNA) of Different OS Hypoxia Subtypes in the TARGET-OS Dataset

The expression matrix of anoxic-related genes in the TARGET-OS dataset was analysed by WGCNA to explore the biological significance of this specific gene using a co-expression relationship in hypoxic environments [29] by employing the WGCNA package [30].

#### 2.1.9 Gene Functional Enrichment Analysis and Pathway Enrichment Analysis

Gene enrichment and pathway annotation of module genes in the OS hypoxia subtype of the TARGET-OS dataset was performed by Gene Ontology (GO) [31] and Kyoto Encyclopedia of Genes and Genomes (KEGG) analyses [32].

#### 2.1.10 Drug Sensitivity Analysis

Drug sensitivity analyses were performed to assist patients with hypoxic subtypes that are not suitable for mRNA vaccine treatment. The Genomics of Drug Sensitivity in Cancer (GDSC) [33] database (www.cancerRxgene.org) was used to predict the sensitivity of OS patients with different OS hypoxia subtypes in the TARGET-OS dataset; the common anticancer drugs or small molecule compounds with IC50 values were evaluated using the Prophetic [34] algorithm. Some core algorithm formulas of this study are provided in supplementary materials.

### 2.2 Clinical Sample and Cell Experiments Validation

This study was approved by the ethics committee of the Children’s Hospital of Chongqing Medical University and was performed in accordance with the Helsinki Declaration (10/01/2023/No. 2022-Y-516) [35]. All of the children's guardians signed a consent form, authorizing the authors to use tissues removed during surgery. Cells lines of 143B were obtained from the Chinese Academy of Sciences. Quantitative real- time PCR was used to evaluate the expression of four tumor antigens [36], and immunohistochemistry was performed on normal bone tissue and osteosarcoma tissue for further verification of our analysis. Transient transfection was used to interfere with four tumor antigen genes [37], and the interference effect was verified by the Western blot experiment. Cell counting kit-8 assay [38] and Transwell assays [39] were used to evaluate the proliferation and invasion ability of cells before and after interference, respectively.

### 2.3 Statistical Analysis

The pseudotime analysis was performed using the R software (version 4.1.1). All other data processing and analyses were based on R software (version 4.2.2). Continuous variables have been displayed as mean ± standard deviation. The Wilcoxon rank sum test was used for comparisons between two groups; the Kruskal‒Wallis test was used for comparisons of three groups and above. If not specified, the correlation coefficients between different molecules were calculated by Spearman correlation analysis, and all statistical *p* values were bilateral, with *p*<0.05 as statistically significant.

## 3 RESULTS

### 3.1 Osteosarcoma-associated Overexpressed Genes

**Fig. (1):**
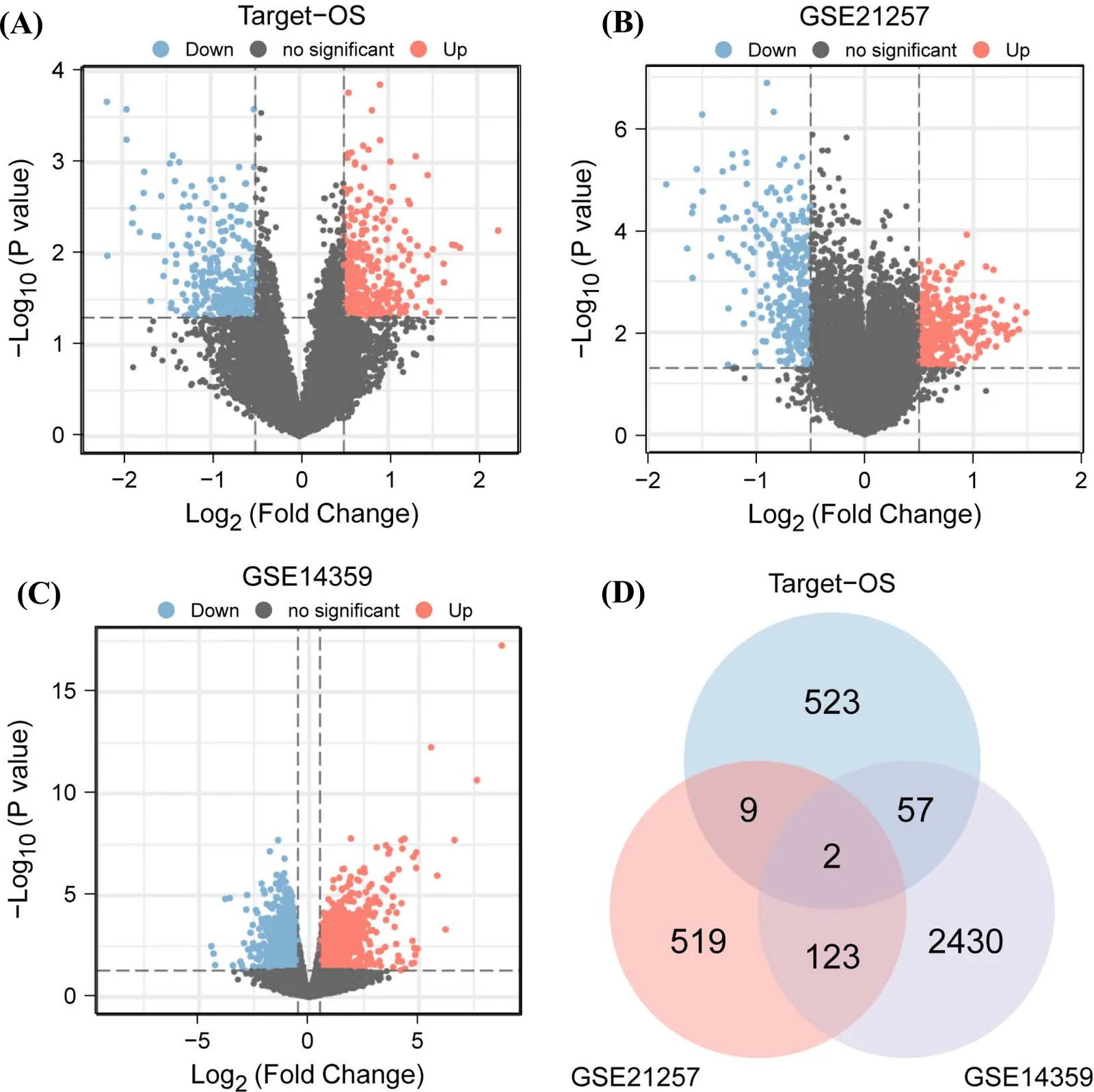
Analysis of OS-associated overexpressed genes. (**A**-**C**). Overexpression gene analysis and volcano plots of metastatic and non-metastatic groups in the TARGET-OS dataset (**A**), GSE21257 dataset (**B**), and GSE14359 dataset (**C**, **D**). Venn diagram of overexpressed genes in the TARGET-OS dataset, GSE21257 dataset, and GSE14359 dataset. OS, osteosarcoma.

Overexpressed genes serve as potential sources of tumor neoantigens, which are crucial for initiating immune responses. Consequently, this study concentrated on identifying such overexpressed genes. A total of 591 overexpressed genes that met the threshold were obtained from the TARGET-OS dataset (Fig. **1A**). 653 genes were overexpressed in the GSE21257 dataset (Fig. **1B**) and 2612 genes were highly expressed in the GSE14359 dataset (Fig. **1C**).

To obtain the overexpressed genes in the OS datasets, the differential genes obtained in the three datasets were intersected. Finally, 191 overexpressed genes from the OS dataset were obtained, and a Venn diagram was plotted (Fig. **1D**).

### 3.2 Prognostic Survival Analysis of Overexpressed Genes

**Fig. (2):**
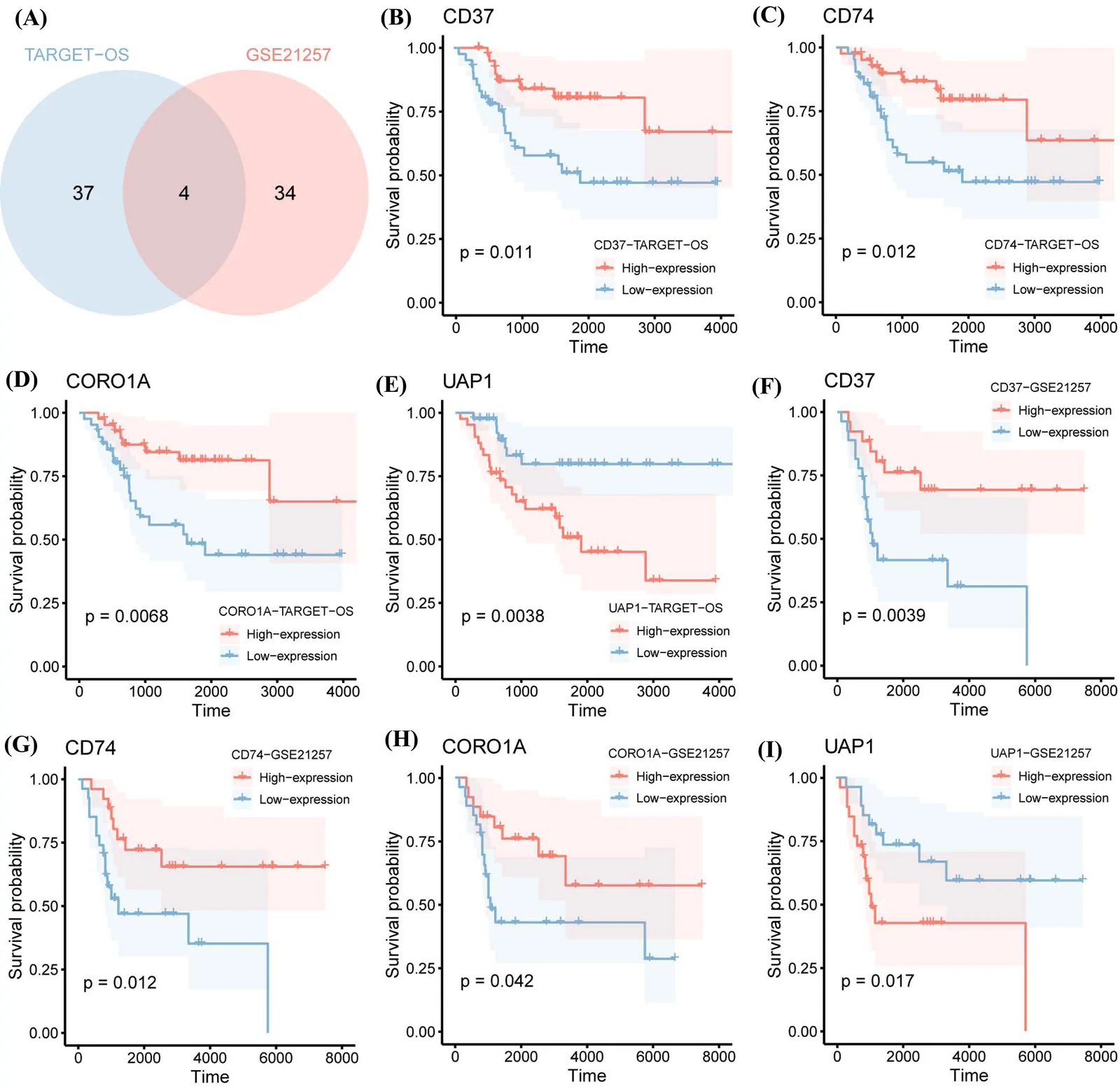
Prognostic KM survival curve analysis (OS) of overexpressed genes in OS datasets. (**A**) Venn diagram presentation of the results of the overall survival as obtained from the KM curve for prognostic analysis of overexpressed genes in TARGET-OS and GSE21257 datasets. (**B**-**E**). The results of KM curves for overexpressed genes CD37 (**B**), CD74 (**C**), CORO1A (**D**), and UAP1 (**E**) in the TARGET-OS dataset. (**F**-**I**). The results of prognostic KM survival curves for overexpressed genes CD37 (**F**), CD74 (G), CORO1A (**H**), and UAP1 (**I**) in the GSE21257 dataset. *p* ≥ 0.05, not statistically significant; *p* < 0.05, statistically significant; *p* < 0.01, highly statistically significant; *p* < 0.001, extremely statistically significant. OS, osteosarcoma; KM, Kaplan‒Meier.

Afterward, the results of the intersection of statistically significant overexpressed genes in the KM curve and the clinical prognosis information of the TARGET-OS and GSE21257 datasets were used to obtain the overexpressed genes in the two datasets, and a Venn diagram was drawn to display the results (Fig. **2A**). We finally obtained four overexpressed genes: CD37, CD74, CORO1A, and UAP1. Then, the results of the KM curve of the prognosis of four overexpressed genes in the TARGET-OS and GSE21257 datasets were acquired (Figs. **2B**-**I**). CD37 (*p*=0.011, Fig. **2B**), CD74 (*p*=0.012, Fig. **2C**), CORO1A (*p*=0.0068, Fig. **2D**), and UAP1 (*p*=0.0038, Fig. **2E**) in the TARGET-OS dataset were significantly associated with the prognosis of OS patients (*p*<0.05) In the GSE21257 dataset, the four selected genes were also significantly associated with the prognosis of OS patients (Figs. **2F**-**I**).

### 3.3 Identification of Potential Hypoxia-related Antigens in OS

Subsequently, we focused on the possibility of these overexpressed genes (CD37, CD74, CORO1A, UAP1) of OS as tumor antigens. Considering the interaction between tumor antigen and antigen-presenting cells, we evaluated the immune infiltration abundance of 10 antigen-presenting cells (endothelial cells, fibroblasts, monocytic lineage cells, myeloid dendritic cells, neutrophils, NK cells, and T cells) in the TARGET-OS dataset by the MCPcounter algorithm. We found CD37, CD74, and CORO1A to be positively correlated with immune cells, while UAP1 was negatively correlated.

Among them, CD37 was highly significantly positively correlated with CD8 T cells, endothelial cells, monocytic lineage, myeloid dendritic cells, and T cells (Figs. **S1A-E**). CD74 was highly significantly positively correlated with endothelial cells, monocytic lineage cells, myeloid dendritic cells, and T cells (Figs. **S1F-I**). CD8 T cells, endothelial cells, monocytic lineage cells, myeloid dendritic cells, and T cells (Figs. **S1J-N**) showed highly significant positive correlations with CORO1A. Monocytic lineage (Fig. **S1O**) showed a highly significant negative correlation with UAP1. These results indicated that the tumor antigens we identified (CD37, CD74, CORO1A, UAP1) may be directly processed by APCs, presented to T cells, and recognized by antigen-presenting cells, thus triggering an immune response.

### 3.4 Identification of a Potential OS Hypoxia Subtype

By identifying different OS hypoxia subtypes of the TARGET-OS dataset by consensus clustering, two OS hypoxia subtypes (cluster 1 and cluster 2) were finally identified (Fig. **S2A**) and both contained 32 samples. The consistent cluster CDF plots and the area under the CDF curve delta plots are shown in Figs. (**S2B, C**). In addition, we performed PCA on the two OS hypoxia subtypes, and PCA clustering showed significant differences between the two OS hypoxia subtype samples (Fig. **S2D**). KM curves (Fig. **S2E**) showed the OS hypoxia subtype (cluster 1/cluster 2) to be highly statistically significant for the diagnosis of prognostic survival outcomes in OS patients (*p* < 0.01), and the OS of hypoxia subtype 1 was significantly worse than that of OS hypoxia subtype 2.

The same HRGs were analysed in the GSE21257 dataset. Two subtypes of OS hypoxia were obtained (Fig. **S2F**). The details of CDF plots are provided in Figs. (**S2G-H**). In addition, PCA clustering results showed the same significant difference between the two OS hypoxia subtype samples in the GSE21257 dataset (Fig. **S2I**). KM curves (Fig. **S2J**) showed the diagnosis of the OS hypoxia subtype to be consistent with the trend of the TARGET-OS dataset.

### 3.5 Expression Differences of ICD- and ICP-regulated Genes in OS Hypoxia Subtypes in OS Datasets

Twenty-five ICD genes were detected in the TARGET-OS dataset (Fig. **S3A**), and 7 genes were differentially expressed (*p* < 0.05) in different OS hypoxia subtypes. A total of 23 ICD genes were detected in the GSE21257 dataset (Fig. **S3B**), and 15 genes were differentially expressed (*p* < 0.05) in different OS hypoxia subtypes.

In addition, 46 ICP genes were detected in the TARGET-OS dataset (Fig. **S3C**), 20 genes were differentially expressed (*p* < 0.05) in different OS hypoxia subtypes, and 40 ICP genes were detected in the GSE21257 dataset (Fig. **S3D**).

We found ICD genes (FPR1, TLR4) to be differentially expressed in both OS datasets and in higher amounts in OS hypoxia subtype 2 patients than in OS hypoxia subtype 1 patients (Figs. **S3A, B**). ICP genes (CD40, HAVCR2, LAIR1, LGALS9, PDCD1LG2, TNFSF4) were differentially expressed in both OS datasets and were significantly less expressed in OS hypoxia subtype 1 patients than in OS hypoxia subtype 2 patients (Figs. **S3C, D**). In conclusion, immunophenotyping could reflect the expression levels of ICD and ICP and could be used as a biomarker for mRNA vaccines. The mRNA vaccine may be less effective in patients with OS hypoxia subtype 2 (ICP, ICD high expression).

### 3.6 Immune Evaluation in OS Datasets and Identification of Immune Infiltrating Cells

**Fig. (3):**
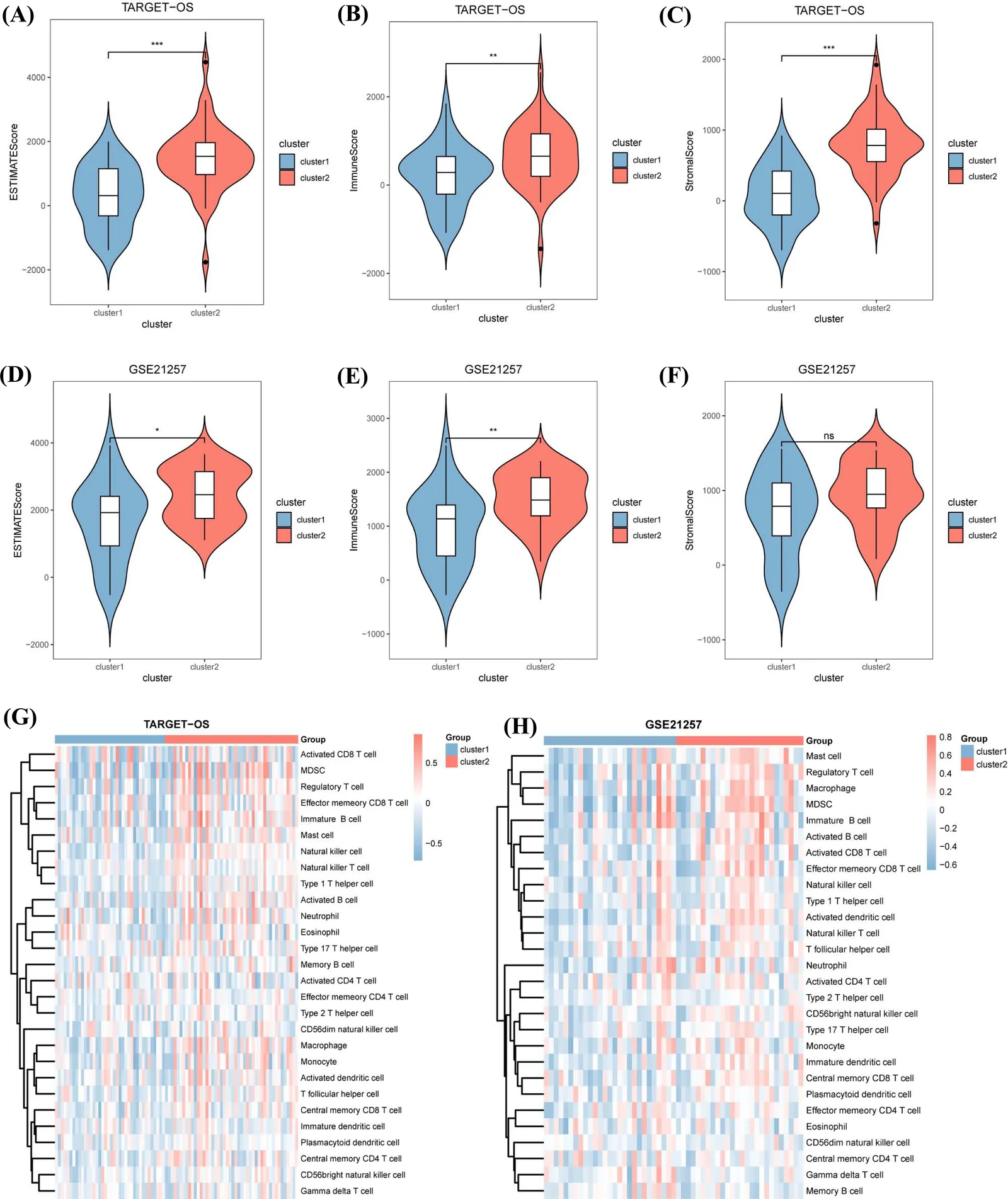
Immune evaluation of OS datasets and identification of immune infiltrating cells. (**A**-**C**). Groupwise comparison plots of ESTIMATEScore (**A**), ImmunoScore (**B**), and StromalScore (**C**) among OS hypoxia subtypes (cluster 1/cluster 2) in the TARGET-OS dataset. (**D**-**F**). Groupwise comparison plots of ESTIMATEScore (**D**), ImmunoScore (**E**), and StromalScore (**F**) among OS hypoxia subtypes (cluster 1/cluster 2) in the GSE21257 dataset. (**G**). Enrichment score heatmap of 28 immune cell infiltration abundances among OS hypoxia subtypes (cluster 1/cluster 2) in the TARGET-OS dataset. (**H**). Enrichment score heatmap of 28 immune cell infiltration abundances among OS hypoxia subtypes (cluster 1/cluster 2) in the GSE21257 dataset. “ns” equates to P≥0.05, not statistically significant; “*” equates to *p* < 0.05, statistically significant; “**” equates to *p* < 0.01, highly statistically significant; “***” equals *p* < 0.001 for extreme statistical significance. OS, osteosarcoma.

The StromalScore and ImmunoScore are components of the ESTIMATE algorithm. These scores are calculated based on the expression levels of specific genes associated with stromal and immune cells within tumor tissues [40]. Figs. (**3A**-**F**) shows the results of the StromalScore, ImmunoScore, and ESTIMATEScore comparison of OS hypoxia subtypes. The results showed the ESTIMATEScore (*p* < 0.001, Fig. **3A**) and StromalScore (*p* < 0.001, Fig. **3C**) of the TARGET-OS dataset to be extremely statistically significant between the different OS hypoxia subtypes. The ImmunoScore (*p* < 0.01, Fig. **3B**) was highly statistically significant between the different OS hypoxia subtypes. The ESTIMATEScore of the GSE21257 dataset (*p* < 0.05, Fig. **3D**) was statistically significant between the different OS hypoxia subtypes, and the ImmunoScore (*p* < 0.01, Fig. **3E**) was highly statistically significant between the different OS hypoxia subtypes. However, the StromalScore (P > = 0.05, Fig. **3F**) was not statistically significant between the different OS hypoxia subtypes.

We found that in both OS datasets, the StromalScore, the ImmunoScore, and ESTIMATEScore were higher in OS hypoxia subtype 2 than in OS hypoxia subtype 1 in both OS datasets (Figs. **3A**-**F**). To explore the differential situation of immune infiltration among different OS hypoxia subtypes in the OS datasets, the ssGSEA algorithm was used to calculate the infiltration abundance of 28 immune cells in different OS hypoxia subtypes. The results are shown in Figs. (**3G**, **H**). The heatmap shows that the abundance of infiltrating immune cells was higher in OS hypoxia subtype 2 (“hot” phenotype) patients than in OS hypoxia subtype 1 (“cold” phenotype) patients in both OS datasets. mRNA vaccines containing these antigens can induce immune infiltration in OS patients in the OS hypoxia subtype 1 group who are immune to “cold”.

### 3.7 Hypoxia Landscape of OS in the TARGET-OS Dataset

**Fig. (4):**
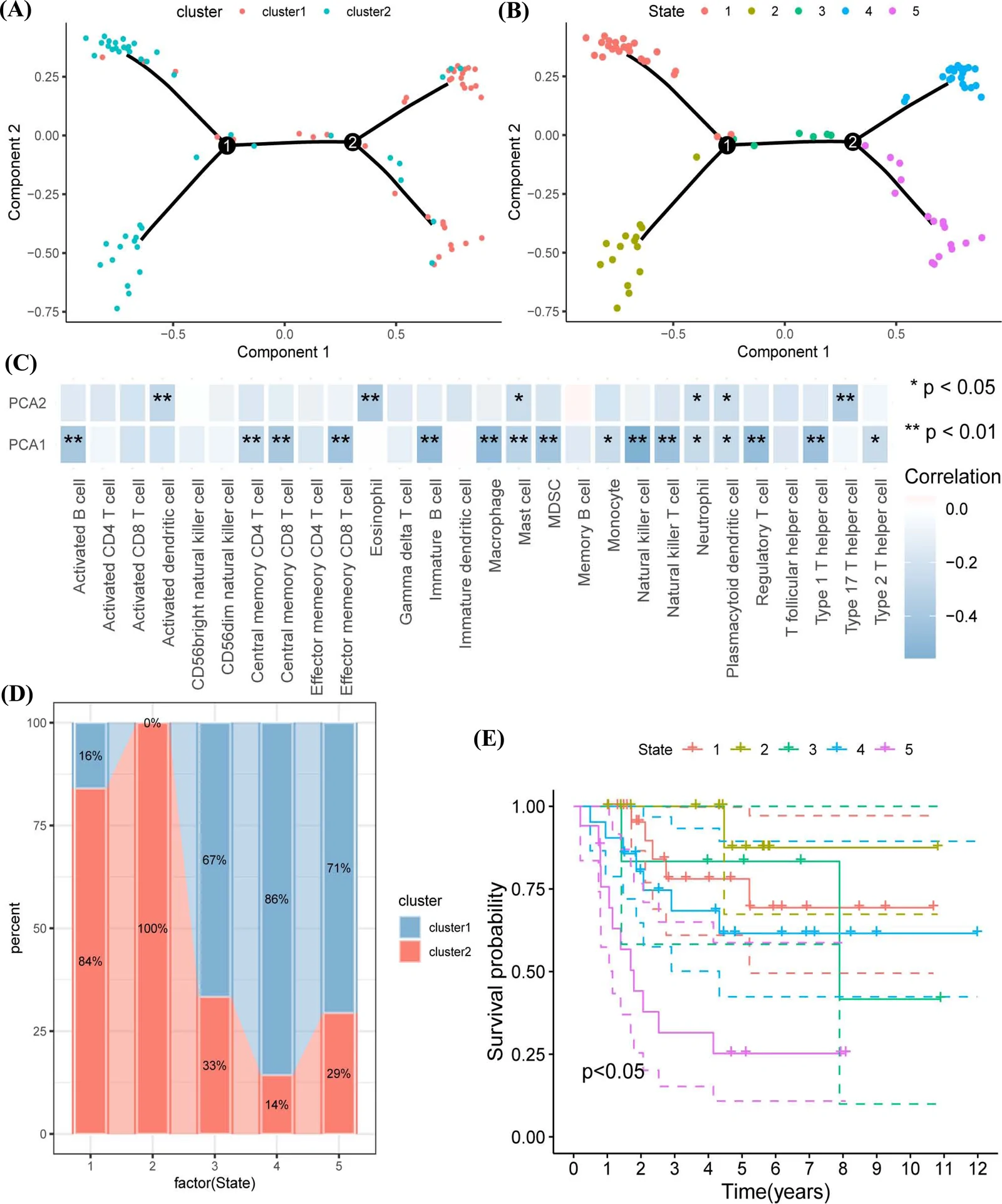
Hypoxia landscape of OS in the TARGET-OS dataset. (**A**) Hypoxia landscape of the TARGET-OS dataset. The location of each OS patient in the immunomap, with color corresponding to the OS hypoxia subtype identified above. (**B**) Hypoxia landscape of the TARGET-OS dataset. OS patients, reclassified according to their location, were divided into states 1, 2, 3, 4, and 5. (**C**) Hypoxia landscape of the TARGET-OS dataset. The results of correlation analysis between PCA 1/2 and 28 immune cells are shown. (**D**) Proportion of different patient OS hypoxia subtypes in the hypoxia landscape of the TARGET-OS dataset. (**E**) Prognostic KM curves for patients at different stages in the hypoxia landscape of the TARGET-OS dataset. *p* ≥ 0.05, not statistically significant; *p* < 0.05, statistically significant; *p* < 0.01, highly statistically significant; *p* < 0.001, extremely statistically significant. **Abbreviations: ** OS, osteosarcoma; KM, Kaplan‒Meier; OS, overall survival.

The hypoxia landscape of OS was constructed by using the expression profile of HRGs of each OS patient in the TARGER-OS dataset (Figs. **4A**, **B**). The OS patients in the OS hypoxia subtypes (cluster 1/cluster 2) were roughly divided into five tracks (Fig. **4B**). Then, we analysed the correlation between the horizontal axis (PCA1) and vertical axis (PCA2) of the hypoxia landscape and the immune infiltration abundance of 28 immune cells obtained by the ssGSEA algorithm and displayed the analysis results through the correlation heatmap (Fig. **4C**). The horizontal axis (PCA1) and vertical axis (PCA2) of the hypoxic landscape were significantly negatively correlated with multiple immune cells, and the negative correlation was relatively more significant between the horizontal axis (PCA1) and the infiltration abundance of multiple immune cells.

The proportion of five state subgroups in different OS hypoxia subtypes in the hypoxia landscape was further analysed. The proportion of these subgroup samples in different OS hypoxia subtypes is shown in Fig. (**4D**). The proportion of the state 1 and state 2 subgroups in OS hypoxia subtype 2 was relatively high, while the proportion of state 3, state 4, and state 5 subgroups in OS hypoxia subtype 1 was relatively high (Fig. **4D**).

Finally, the KM curve of the five state subgroups was drawn (Fig. **4E**). The prognosis analysis showed that the overall survival curves of the five states had significant differences, among which the prognosis of state 5 was the worst and that of state 2 was the best (Fig. **4E**). In summary, the immune map based on the OS hypoxia subtype could better identify the immune status of each OS patient and predict its prognosis, and can be conducive to the personalized treatment choice of mRNA vaccines.

### 3.8 WGCNA Identified Hypoxia Gene Coexpression Modules in the TARGET-OS Dataset

In the WGCNA, OS samples were clustered by a clustering tree (Fig. **S4A**), and the optimal power threshold was subsequently set at a screening criterion of 0.85. As can be seen in Fig. (**S4B**), the optimal power threshold at which we performed WGCNA was 7. HRGs were clustered into 7 modules (Fig. **S4C**). HRGs were clustered, and the relationship between HRGs and corresponding modules was visualized (Fig. **S4D**). Finally, the correlation between the 7 modules and OS hypoxia subtypes was obtained (Fig. **S4E**).

A total of 2 modules, MEyellow (|COR|=0.4, *p*=2e-04) and MEturquoise (|COR|=-0.3, *p*=0.005), with statistically significant differences between hypoxia subtypes of OS in the TARGET-OS dataset, were selected, and a total of 177 hypoxia-related module genes (HRMGs) included in the two modules were subjected to subsequent analysis. The genes (Table **S3**) in these important modules may serve as biomarkers for predicting the prognosis of OS patients and may identify suitable patients for mRNA vaccines.

The subsequent analysis of WGCNA modules between different OS hypoxia subtypes revealed that in multiple modules, OS hypoxia subtype 1 scored significantly higher than OS hypoxia subtype 2, and the MEgreen, MEturquoise, and MEyellow modules had statistically significant differences among different OS hypoxia subtypes (*p* < 0.05) (Fig. **S4F**). We also performed multivariate Cox analysis for the 7 modules in the WGCNA results, but found that no module was able to serve as an independent prognostic factor (Fig. **S4G**).

### 3.9 Functional Enrichment Analysis and Pathway Enrichment Analysis of HRMGs

**Fig. (5):**
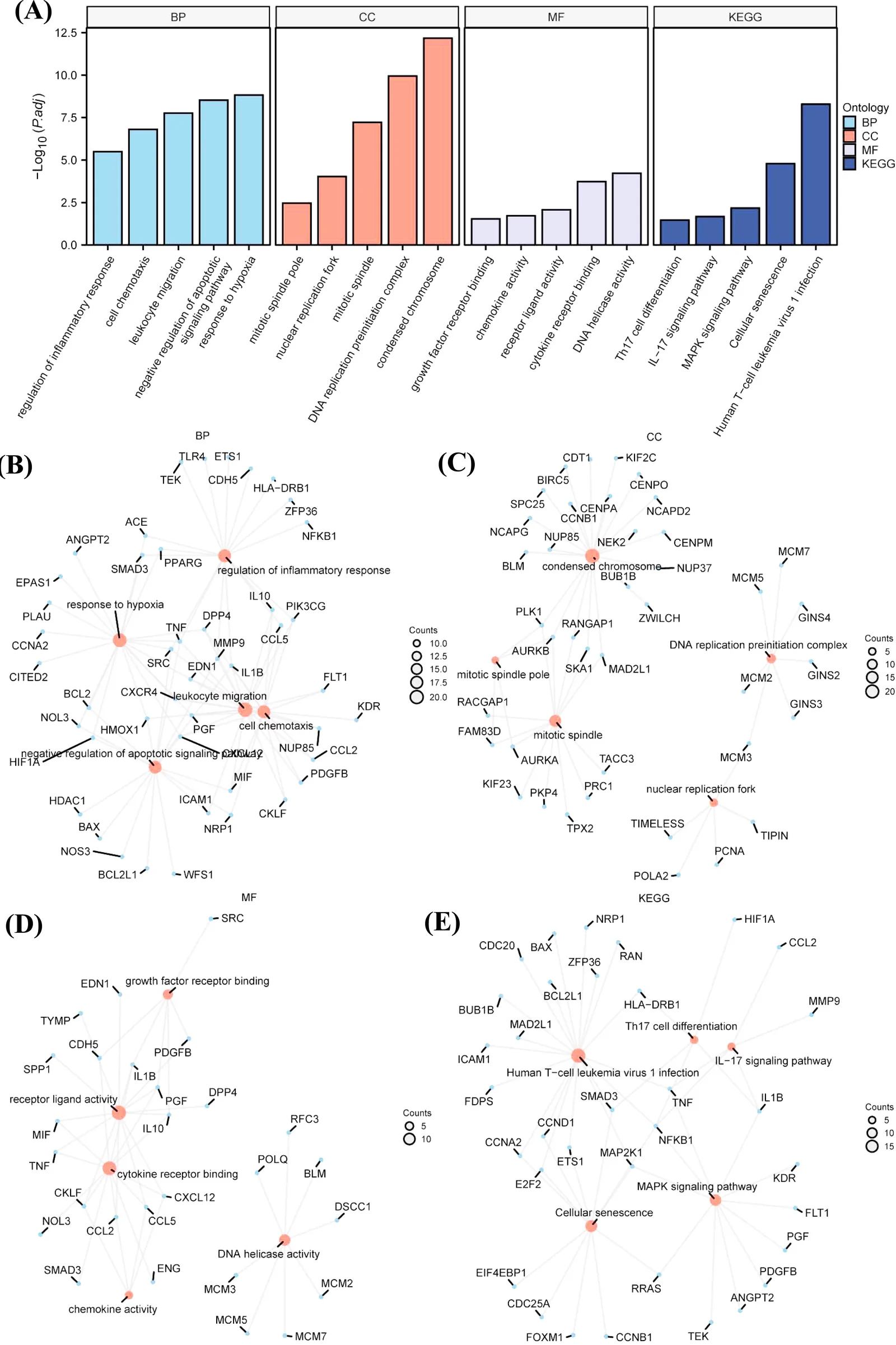
Functional enrichment analysis and pathway enrichment analysis of HRMGs. (**A**) GO functional enrichment analysis and KEGG pathway enrichment analysis of HRMGs are displayed in the form of a bar chart. (**B**-**D**) GO functional enrichment analysis of the HRMG network diagram displays the BP results (**B**), CC results (**C)**, and MF results (**D**). E. Network plot presentation of KEGG pathway enrichment analysis results of HRMGs. The abscissa in the histogram (**A**) indicates GO terms, the ordinate refers to the p.adj size of GO terms, and the color indicates different categories, with dark cyan representing the BP pathway, orange-red representing the CC pathway, light purple representing the MF pathway, and indigo cyan representing the KEGG pathway. Dark cyan dots in network plots (**B**-**E**) represent specific genes, and orange-red circles represent specific pathways. **Abbreviations: **HRMGs, hypoxia-related module genes; GO, Gene Ontology; BP, biological process; CC, cellular component; MF, molecular function; KEGG, Kyoto Encyclopedia of Genes and Genomes. The screening criteria of GO and KEGG enriched entries were p.adj < 0.05 and FDR value (q.value) < 0.05.

To analyse the relationships among BP, MF, CC, biological pathway, and OS of 177 HRMGs in different OS hypoxia subtypes of the TARGET-OS dataset, first, 177 HRMGs were subjected to GO functional enrichment analysis and KEGG pathway enrichment analysis (Table **S4**). The results showed the 177 HRMGs to be mainly enriched in BP, such as response to hypoxia, and CC, such as condensed chromosome; they were also enriched in MF, such as DNA helicase activity, and additionally in KEGG pathways, such as human T-cell leukemia virus 1 infection. We demonstrated the results using bar plots (Fig. **5A**). In addition, we have also demonstrated the BP, CC, and MF pathway enrichment results of GO gene functional enrichment analysis in the form of network plots (Figs. **5B**-**D**), and the KEGG pathway enrichment analysis results have been similarly exhibited in the form of ring network plots (Fig. **5E**).

### 3.10 Drug Sensitivity Analysis of the TARGET-OS Dataset with Respect to Drugs given to Patients with Different OS Hypoxia Subtypes

Drug sensitivity analysis was performed to explore the therapeutic strategies suitable for patients with hypoxic subtypes that are not suitable for mRNA vaccine treatment. The top 20 drugs with relatively large differences in the different OS hypoxia subtypes were subsequently retained (Figs. **S5A-T**). We found that among the 20 drugs with significant differences, drug sensitivity was higher in patients with prevalent OS hypoxia subtype 2 than in those with OS hypoxia subtype 1 (Figs. **S5A-T**). It was speculated based on the above results that patients with OS hypoxia subtype 2 might have a higher sensitivity to these agents, which also further emphasized the importance of individualized treatment for tumor patients.

### 3.11 Clinical Sample and Cell Experiments Validation

**Fig. (6):**
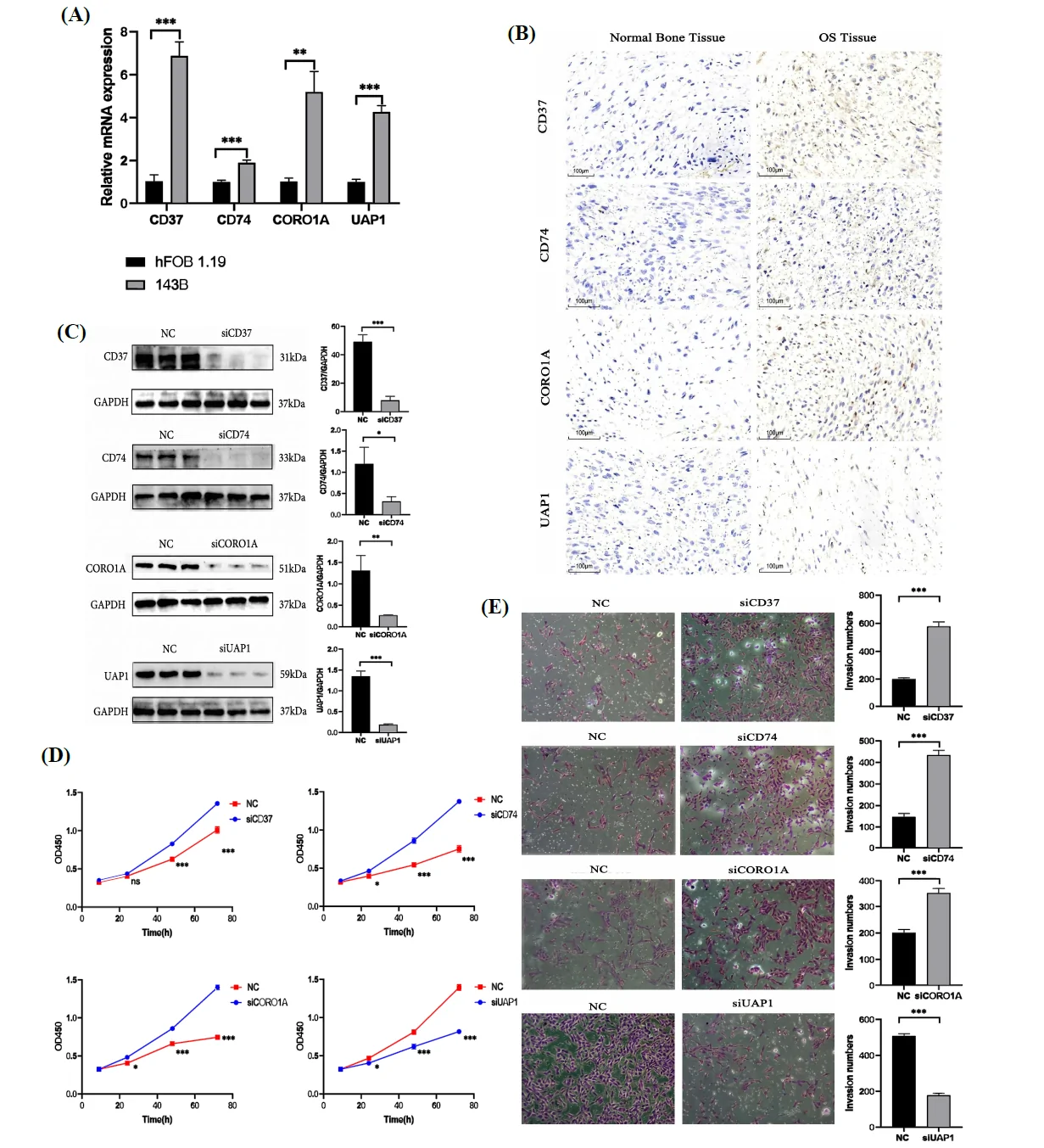
Clinical sample validation. (**A**) The expression level of CD37, CD74, CORO1A, and UAP1 in osteosarcoma cells was detected by quantitative real-time PCR. (**B**) The expressions of CD37, CD74, CORO1A, and UAP1 in OS tissues and normal bone tissues were detected by the immunohistochemical method. (**C**) Western blot detected the knockdown of CD37, CD74, CORO1A, and UAP1 in 143B cell lines. (**D**) The proliferation of OS cells was detected by CCK-8. (**E**) The invasion of 143B was detected by Transwell assay. Data (mean ± SEM) represent three independent experiments. “ns” equates to *p*≥0.05, not statistically significant; “*” equates to *p* < 0.05, statistically significant; “**” equates to *p* < 0.01, highly statistically significant; “***” equals *p* < 0.001 for extreme statistical significance. OS, osteosarcoma.

Quantitative real-time PCR further confirmed the four tumor antigens to be highly expressed at the mRNA level in osteosarcoma cells (Fig. **6A**). At the same time, immunohistochemistry was performed on osteosarcoma tissues and normal bone tissues of clinical samples (Fig. **6B**).

To further verify the potential impact of these four tumor antigens in OS cells, we knocked down the expression of these four tumor antigen genes in the 143B cell line by RNA interference technology and then used CCK-8 and Transwell assays to detect the impact of these four tumor antigens on 143B proliferation and invasion (Fig. **6C**). The results showed that the knockdown of these four tumor antigen genes in OS cells could affect the proliferation rate and invasive ability of the cells (Figs. **6D**, **E**). In summary, the data indicated that four tumor antigens identified could be considered potential antigens for anti-OS mRNA vaccines.

## 4 DISCUSSION

As discovered in recent decades, metastatic disease is still present in nearly one-quarter of patients, making metastasis the major cause of death in OS patients [2-4]. Therefore, novel therapeutic approaches urgently need to be explored to further improve the prognosis of OS patients.

The development of mRNA vaccine technology in recent years has provided us with a new idea and solution [20, 21, 41, 42]. The mRNA vaccine is a nucleic acid-based vaccine that involves the introduction of artificially synthesized mRNA molecules into human cells to induce the production of vaccine antigen proteins, thereby eliciting an immune response against specific pathogens [43]. Compared to traditional vaccines, mRNA vaccines have a shorter development cycle, lower research costs, and higher immune protection and safety [44].

Although mRNA vaccine technology has shown excellent performance in COVID-19 prevention and control [45], its potential has not been fully explored in research on other diseases. For instance, treating a specific disease may require the selection of suitable targets and antigens based on its pathogenic mechanism, but the screening process for these targets and antigens is complex and challenging.

Therefore, further research and development of new mRNA vaccine technologies are needed to better address the challenges of various diseases. For unexplored applications of mRNA in a particular disease, researchers can use high-throughput technologies and bioinformatics methods to find suitable targets and antigens and continuously optimize mRNA vaccine technology to improve its efficacy and safety.

There are two core issues that need to be addressed in the field of tumor mRNA therapy. The first step is to construct mRNA sequences for specific antigens. In order to achieve specific elimination of tumor cells, the corresponding antigen should be highly expressed in tumor tissue and rarely expressed in normal tissue. This requires the use of high-throughput sequencing data for screening and clinical validation [46]. At the same time, the screening process for potential antigens in mRNA vaccines may require more high-quality algorithms, especially clustering analysis algorithms, to ensure that we can find sufficiently accurate potential antigens [25]. Another issue is how to enable mRNA vaccines to smoothly activate immune responses. Tumor antigens denote distinct antigens manifested within neoplastic cells, encompassing tumor-specific antigens arising from mutagenic genetic alterations or tumor-associated antigens resulting from exaggerated expression of normal cell antigens. Immunopresenting cells, including dendritic cells, macrophages, and B cells, possess the capacity to discern and process these antigens, followed by their presentation to T cells *via* major histocompatibility complex (MHC) molecules [47]. A common strategy is to evaluate the immune level of patients, select populations with high immune response levels, and utilize potential ICD and ICP genes to activate immune responses to mRNA vaccines [48, 49]. This study also used a similar strategy to explore the possibility of mRNA vaccines for osteosarcoma.

The study aimed to identify potential antigens for developing mRNA vaccines to treat a specific disease. An aberrant expression and genomic alteration landscape were constructed to reveal a broad range of potent antigenic targets. To confirm the clinical relevance of the selected antigens, prognostic analysis and immune correlations were conducted. Candidate antigens were identified based on their association with poor prognosis, high infiltration of antigen-presenting cells, and high level of MHC class II gene expression. These antigens play critical roles in the development and progression of the disease and can be presented directly to CD8^+^ T cells to induce an immune response in the presence of adequate lymphocyte infiltration. The potential for mRNA development of these candidates is supported by previous reports, although they have yet to be functionally validated. This study is the first to screen for potential antigens CD37, CD74, CORO1A, and UAP1 for developing mRNA vaccines to treat OS.

Aberrant expression and genomic alterations of various genes were identified in OS patients in this study, which revealed potential targets for developing therapeutic interventions. For example, CD74 was identified as a promising mRNA vaccine candidate for breast cancer, with its upregulation associated with poor tumor prognosis and progression [50, 51]. Meanwhile, CORO1A in breast cancer is also considered to be closely related to clinicopathological factors [52, 53]. In addition, UAP1 was identified as a potential target for developing therapeutic interventions in prostate cancer [54]. CD37 is also considered a potential target for the treatment of acute myeloid leukaemia [55]. The functions of these 4 genes were shown to be involved in DNA damage tolerance, tumor cell survival, apoptosis resistance, and cancer cell proliferation, among others [56]. Furthermore, posttranslational modifications, such as glycosylation, phosphorylation, and acetylation, may also generate tumor antigens, which deserve more in-depth study for future tumor antigen identification [57-59].

Considering that hypoxia is an important factor affecting the proliferation and invasion of tumor cells in the process of tumor development, and the role of hypoxia in the progression of OS is also very important, we determined hypoxia-related antigen as a candidate for the development of an anti-OS mRNA vaccine [60-62]. This provided a theoretical basis for developing anti-OS mRNA vaccines to improve the prognosis of OS patients. This paper has discussed the identification of hypoxia subtypes in OS patients, which can help in selecting the appropriate population for mRNA vaccine therapy. The study identified two hypoxia subtypes based on hypoxia gene expression profiles, including OS hypoxia subtype 1 and OS hypoxia subtype 2, each exhibiting different molecular, cellular, and clinical features [63]. Compared to OS hypoxia subtype 2, OS hypoxia subtype 1 tumor patients have been found to have a better prognosis. The hypoxia subtype has also been found to indicate the therapeutic response to the mRNA vaccine. The study suggested that OS hypoxia subtype 1 tumor patients with high TMB and somatic mutation rates may have a higher response to mRNA vaccines, while OS hypoxia subtype 2 tumors may have an immunosuppressive tumor microenvironment. Our study identified hub genes negatively correlated with immune responses, which may contribute to the development of personalized vaccines. The authors suggest that mRNA vaccines combined with ICP or ICD modulators may revive the immune system and increase immune cell infiltration in immune cold and immune-suppressive tumors [64, 65]. Finally, OS hypoxia subtype 1 tumors have been found to show a good immune activation phenotype, which may respond to ICP. Overall, this study has highlighted the potential for personalized immunotherapy strategies in OS patients based on their hypoxia subtype and tumor immune microenvironment [66].

It must be pointed out that for some patients who cannot respond well to immune responses, mRNA vaccines may be ineffective [9]. Therefore, our drug sensitivity analysis was aimed at helping patients who are not sensitive to mRNA vaccines [67, 68].

However, the present study also involved certain limitations. Although clinical samples and cell experiments have been used for validation, as a retrospective study, the hypoxia-related antigens of the anti-OS mRNA vaccine identified in our study need to be validated in future studies [69]. This includes verifying the role of the hypoxia-related antigen of anti-OS mRNA vaccine in animal models, and further differentiating the hypoxia subtypes of OS patients through relevant clinical trials, to further determine the prognosis of patients with different hypoxia subtypes of OS [70]. In addition, since this study has taken the first step to treat OS with mRNA vaccine, we first determined the hypoxia-related antigens of the anti-OS mRNA vaccine and patients with hypoxia subtypes of OS suitable for vaccination with mRNA vaccine; hence, this study did not involve the specific synthesis and delivery process of mRNA vaccine [71]. If this study is recognized, we will try to use lipid nanoparticles to synthesize and deliver mRNA vaccines in the future [72-74], and further verify the role of mRNA vaccines in the treatment of OS through animal experiments and clinical trials.

This study aimed to identify hypoxia-related antigens for developing anti-OS mRNA vaccines and distinguish between different OS hypoxia subtypes in OS patients. We used bioinformatics data analysis to identify potential antigens, defined OS hypoxia subtypes, and characterized the tumor immune microenvironment of the cancer type. The hypoxia-related antigen of the anti-OS mRNA vaccine identified in this study provided a theoretical basis for the development of anti-OS mRNA vaccines. The establishment of the hypoxia subtypes not only provided a reference for the prognosis of OS patients, but can also help select appropriate OS patients for mRNA vaccine treatment. Overall, this study, as the first step in the treatment of OS with an mRNA vaccine, has laid a practical and reliable theoretical foundation for the development of an anti-OS mRNA vaccine.

## CONCLUSION

This study has identified potential antigens for the anti-OS mRNA vaccine as well as different OS hypoxia subtypes, guiding more effective immunotherapeutic strategies and selecting appropriate patients for tumor vaccine therapy.

## Figures and Tables

**Table 1:** OS dataset information.

-	**TARGET-OS**	**GSE21257**	**GSE14359**
Platform	TARGET	GPL10295	GPL96
Species	Homo sapiens	Homo sapiens	Homo sapiens
Tissue	OS tumor tissues	OS tumor tissues	OS tumor tissues
Samples in non-metastatic group	63	19	10
Samples in metastatic group	21	34	8
Reference	/	[13]	[14]

**Abbreviation: **OS, osteosarcoma.

## Data Availability

The data and supportive information are available within the article.

## LIST OF ABBREVIATIONS

HRGs: Hypoxia-related Genes
ICPs: Immune Checkpoint Proteins
ICD: Immunogenic Cell Death
MSigDB: Molecular Signatures Database
KM: Kaplan‒Meier
GO: Gene Ontology
KEGG: Kyoto Encyclopedia of Genes and Genomes
