# Supplementary material for: Identification and Experimental Validation of Tumor Antigens and Hypoxia Subtypes of Osteosarcoma for Potential mRNA Vaccine Development
Source: Curr Med Chem. 2025 Apr 29;33(15):2951–68. doi: 10.2174/0109298673348041250407062346 (PMC13555841; doi:10.2174/0109298673348041250407062346)
Supplement: Supplementary file 1 [file CMC-33-15-2951_SD1.pdf]

Supplementary Material

Identification and Experimental Validation of Tumor Antigens and Hypoxia Subtypes of Osteosarcoma for Potential mRNA Vaccine Development

Chunnian Ren[1,3], Dawei He[2] and Quan Wang[1,*]

[1]Department of Cardiothoracic Surgery, Children's Hospital of Chongqing Medical University, National Clinical Research Center for Child Health and Disorders, Ministry of Education Key Laboratory of Child Development and Disorders, China International Science and Technology Cooperation Base of Child Development and Critical Disorders, Chongqing Key Laboratory of Pediatrics, Chongqing, PR China; [2]Department of Urology Surgery, Children's Hospital of Chongqing Medical University, Chongqing, PR China; [3]Department of Pediatric Surgery, Chengdu Women's and Children's Central Hospital, School of Medicine, University of Electronic Science and Technology of China, Chengdu, 611731, China

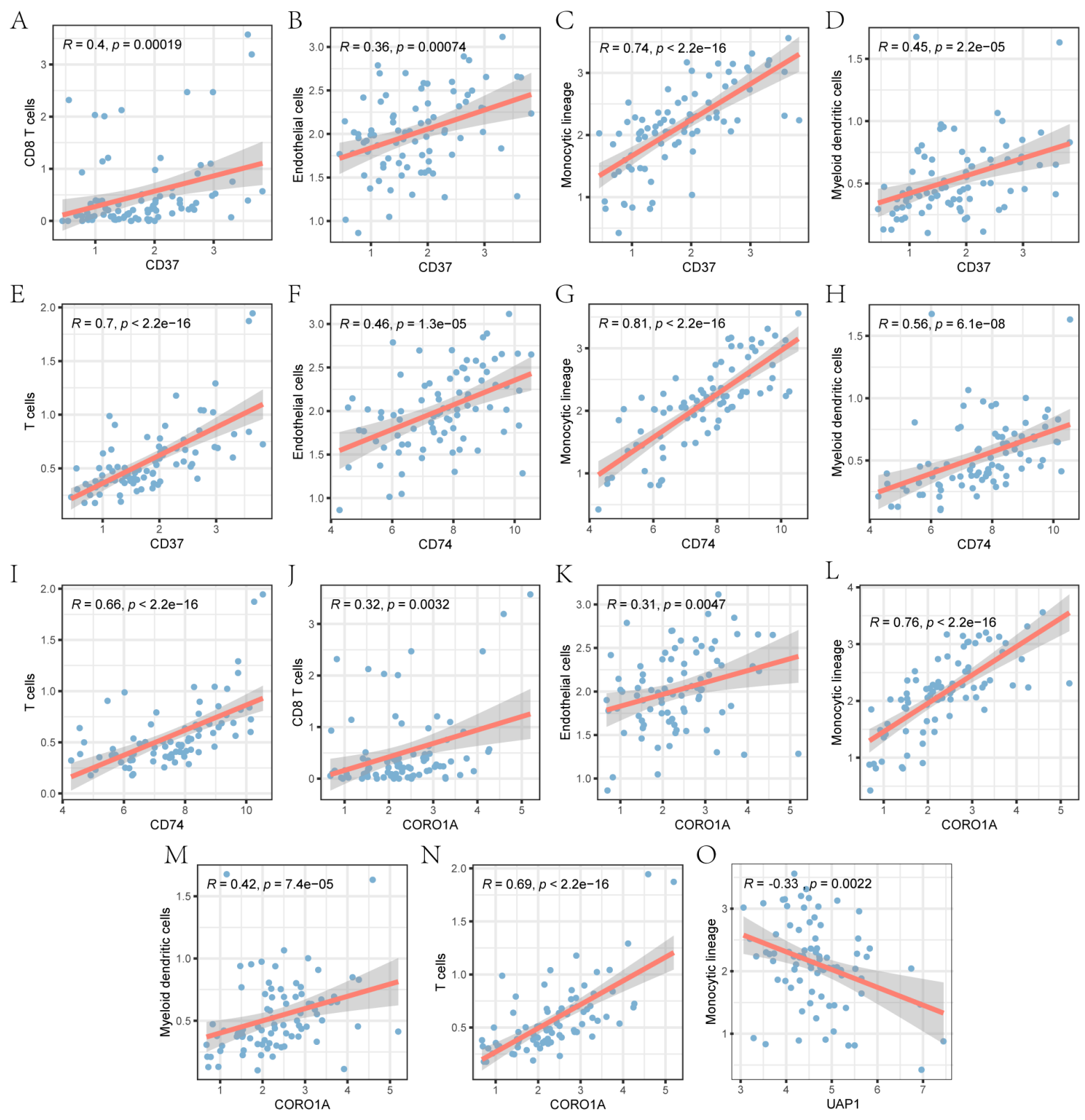

Fig. (S1). Potential immune-related antigen identification in OS. A-E. Scatter plots displaying the results of correlation analysis between overexpressed gene CD37 and antigen-presenting cells CD8 T cells (**A**), Endothalia cells (**B**), Monocytic lineage (**C**), Myeloid dendritic cells (**D**), T cells (**E**). F-I. Scatter plots displaying the results of correlation analysis between overexpressed gene CD74 and antigen-presenting cells Endothalia cells (**F**), Monocytic lineage (**G**), Myeloid dendritic cells (**H**), and T cells (**I**). J-N. Scatter plots displaying the results of correlation analysis between overexpressed gene CORO1A and antigen-presenting cells CD8 T cells (**J**), Endothalia cells (**K**), Monocytic lineage (**L**), Myeloid dendritic cells (**M**), and T cells (**N**). O. Scatter plots displaying the results of correlation analysis between the overexpressed gene UMP1 and antigen-presenting cells of the monocytic lineage. $P \geq 0.05$, not statistically significant; $P < 0.05$, statistically significant; $P < 0.01$, highly statistically significant; $P < 0.001$, extremely statistically significant. Correlation coefficient (**R**) absolute values above 0.8 in the correlation scatter plot are strongly correlated; moderately correlated at absolute values of 0.5-0.8; absolute values at 0.3-0.5 are weakly correlated; and absolute values below 0.3 are weak or uncorrelated. OS, osteosarcoma.

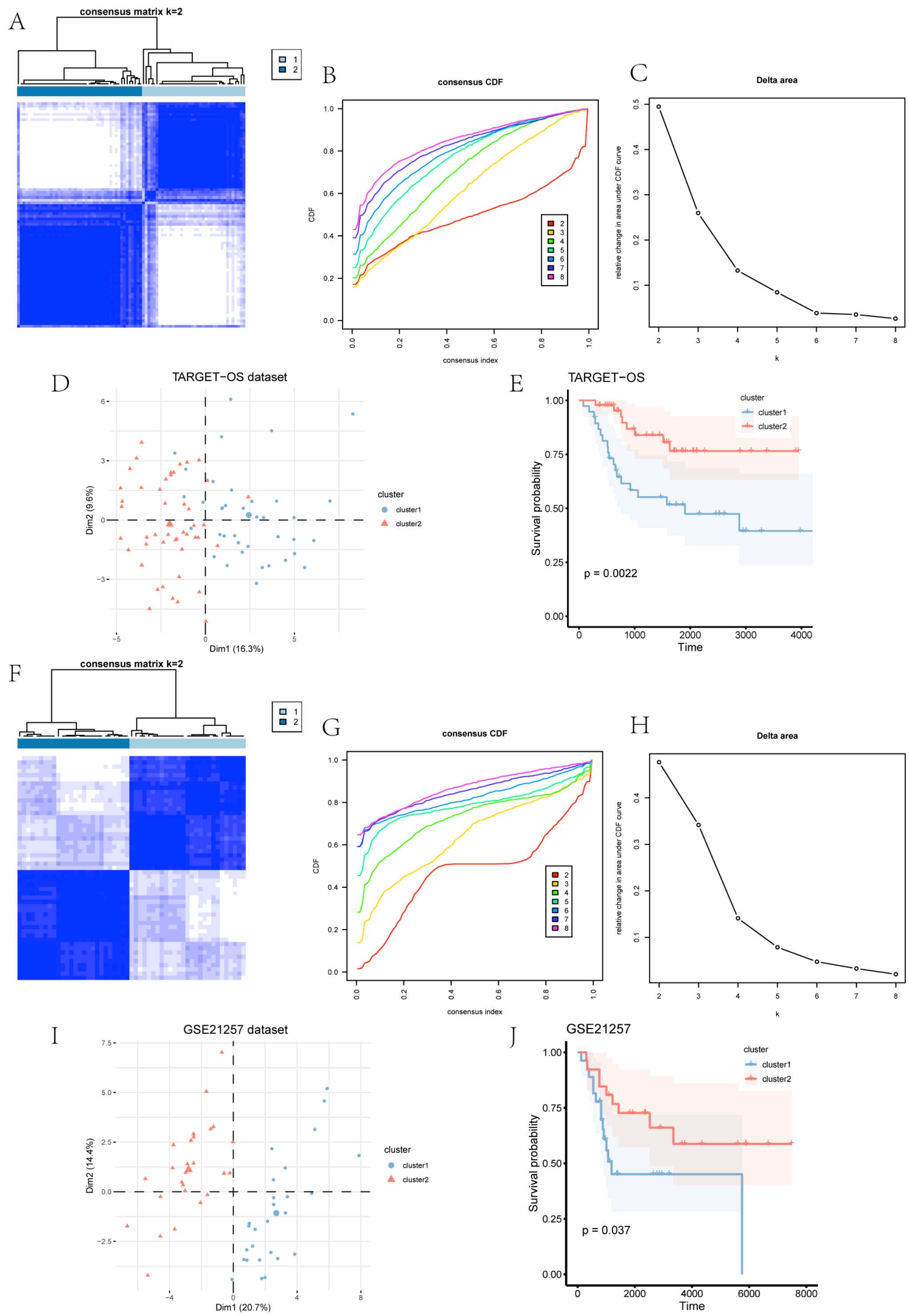

Fig. (S2). Construction of potential OS hypoxia subtypes in OS. (**A**). Consensus clustering (k = 2) result plots of HRGs in the TARGET-OS dataset. B-C. Consistent cluster CDF plot (**B**), area under the CDF curve delta plot (**C**) of different cluster numbers in the consensus clustering of the TARGET-OS dataset. D-E. The results of PCA of the two OS hypoxia subtypes (cluster 1 and cluster 2) of the TARGET-OS dataset are presented (**D**), and the results of prognostic KM curve analysis are presented (**E**). F. Consensus clustering (k = 2) result plots of HRGs in the GSE21257 dataset. G-H. Consistent cluster CDF plot (**g**), area under the CDF curve delta plot (**H**) of different cluster numbers in the GSE21257 dataset consensus clustering. I-J. The results of PCA of the two OS hypoxia subtypes (cluster 1 and cluster 2) of the GSE21257 dataset are presented (**I**), and the results of prognostic KM curve analysis are presented (**J**). $P \geq 0.05$, not statistically significant; $P < 0.05$, statistically significant; $P < 0.01$, highly statistically significant; $P < 0.001$, extremely statistically significant. OS, osteosarcoma; CDF, cumulative distribution function; PCA, principal component analysis; HRGs, hypoxia-related genes.

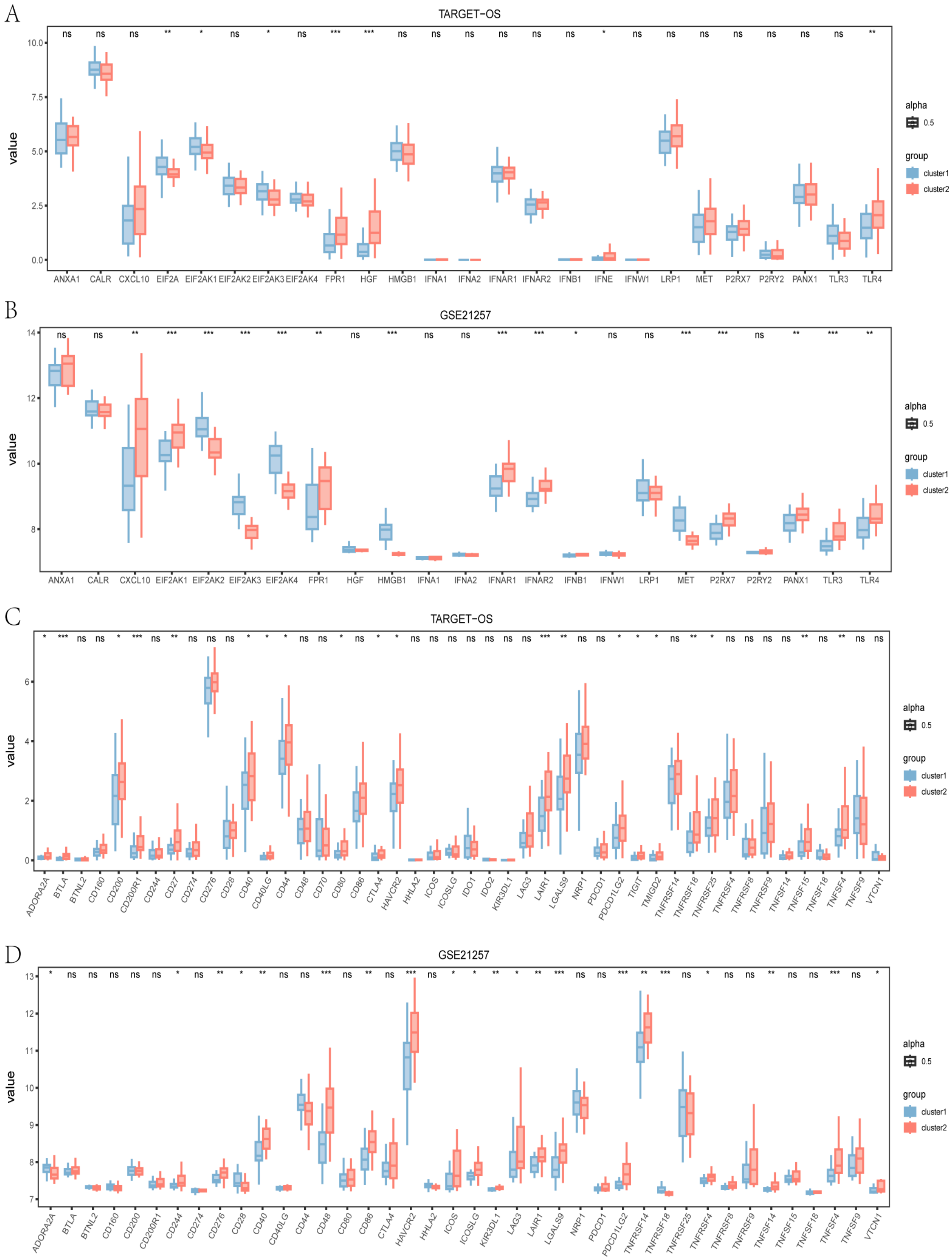

Fig. (S3). Expression differences in ICD- and ICP-regulated genes in OS hypoxia subtypes in OS datasets. A-B. Grouped expression differences of ICD regulators between the TARGET-OS dataset (**A**) and GSE21257 dataset (**B**) OS hypoxia subtypes. C-D. Grouped expression differences in ICP regulators between the TARGET-OS dataset (**C**) and GSE21257 dataset (**D**) OS hypoxia subtypes. "ns" indicates $P \geq 0.05$, not statistically significant; "*" indicates $P < 0.05$, statistically significant; "**" indicates $P < 0.01$, highly statistically significant; "***" indicates $P < 0.001$ for extreme statistical significance. OS, osteosarcoma; ICD, immunogenic cell death; ICP, immune checkpoint protein.

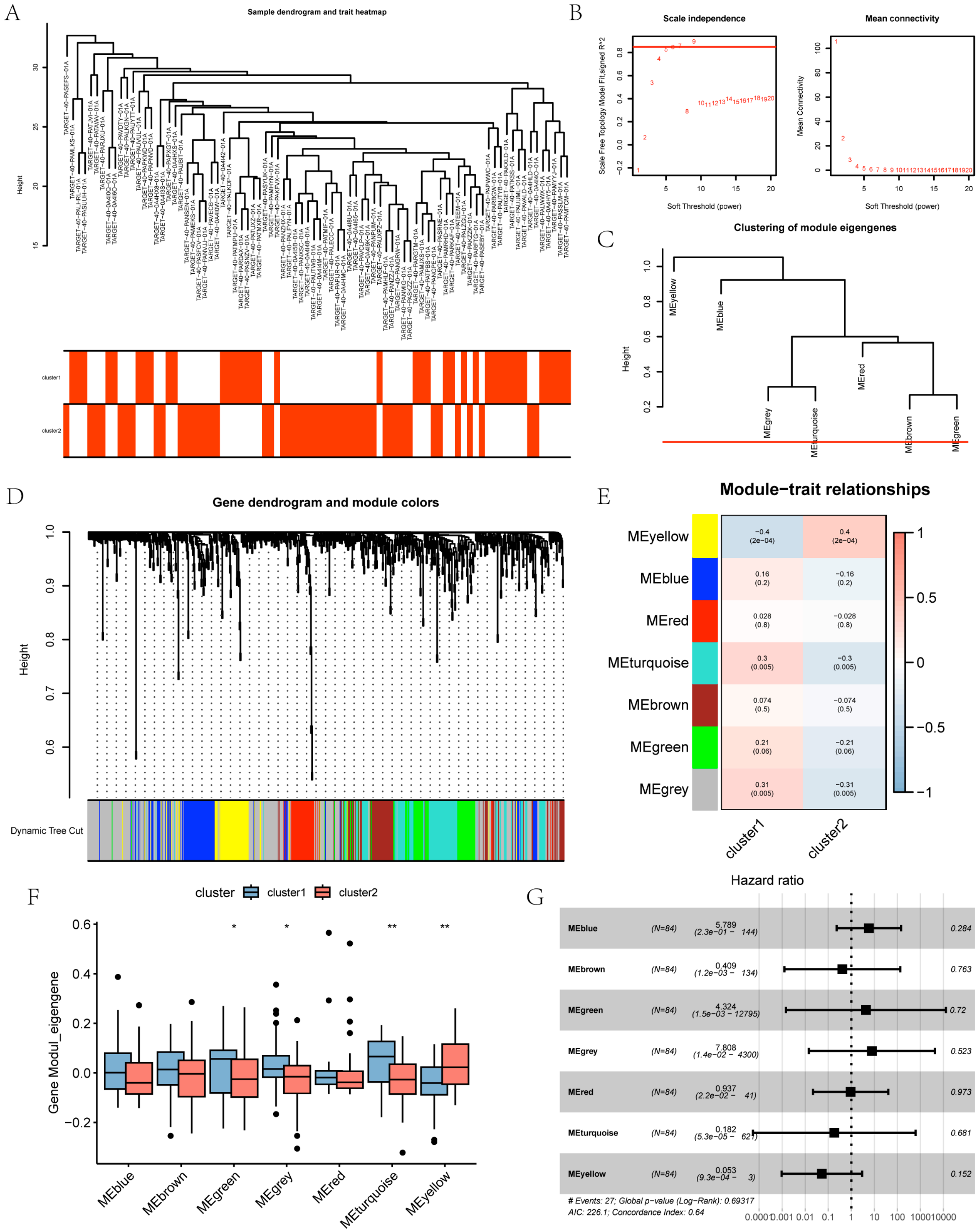

Fig. (S4). WGCNA identifies hypoxia gene coexpression modules in the TARGET-OS dataset. (**A**). Sample clustering result presentation in the TARGET-OS dataset. (**B**). Sample module screening threshold scale-free network display in the TARGET-OS dataset. C. Gene module aggregated result displayed in the TARGET-OS dataset. (**D**. HRGs and module correspondence displayed in the TARGET-OS dataset. (**E**). The results of correlation analysis between the TARGET-OS dataset gene clustering module and the hypoxia subtype of OS are shown. (**F**). Groupwise comparison plot results of different module scores among different OS hypoxia subtypes in the TARGET-OS dataset are shown. (**G**). The results of multivariate Cox analysis of different module scores of the TARGET-OS dataset are presented. "ns" equates to $P \geq 0.05$, not statistically significant; "*" equates to $P < 0.05$, statistically significant; "**" equates to $P < 0.01$, highly statistically significant; " ***" equals $P < 0.001$ for extreme statistical significance. OS, osteosarcoma; WGCNA, weighted gene coexpression network analysis; HRGs, hypoxia-related genes.

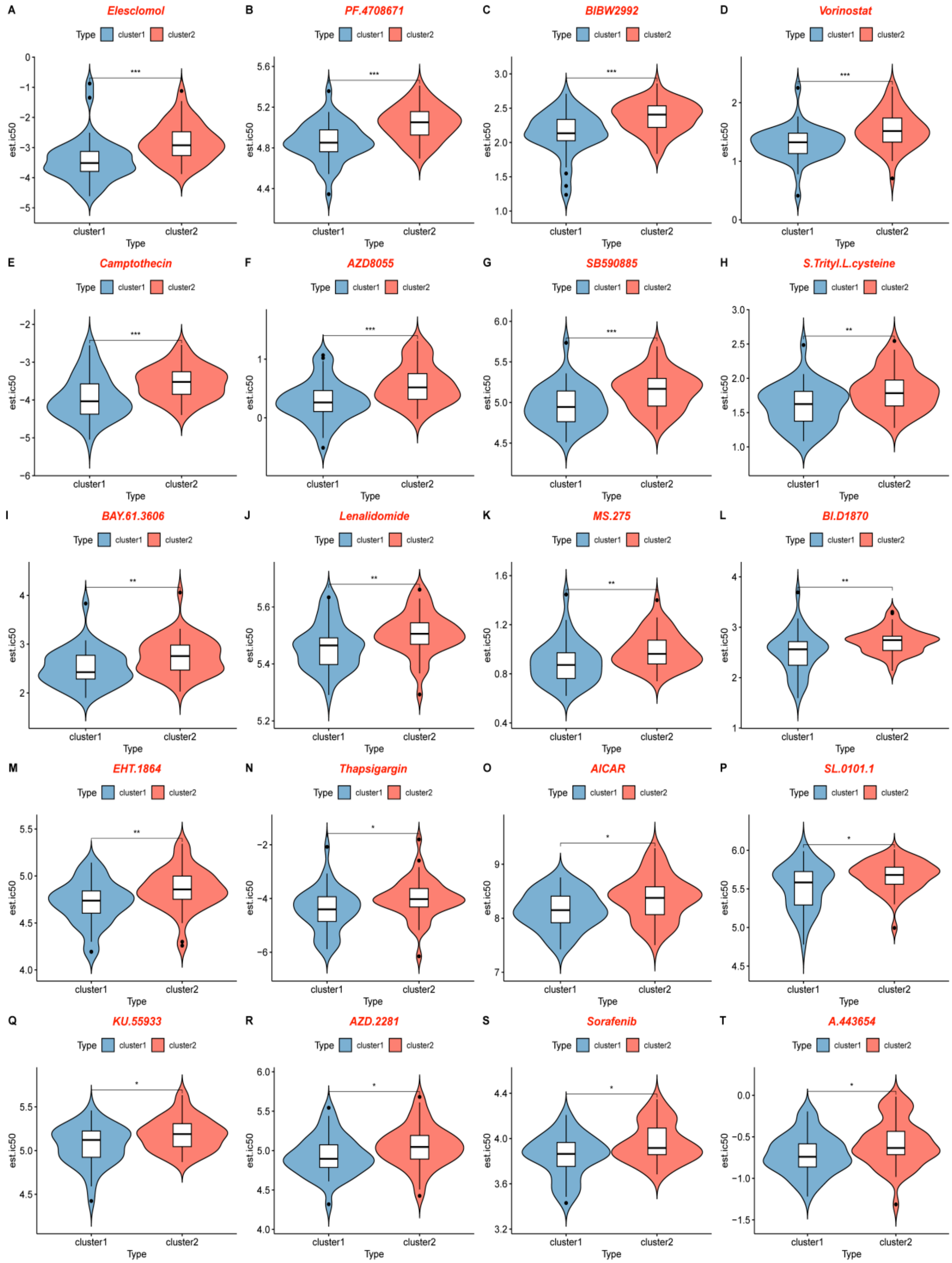

Fig. (S5). Sensitivity analysis to drugs in patients with different OS hypoxia subtypes in the TARGET-OS dataset. A-T. (**A**) groupwise comparison plot demonstrating the results of the sensitivity analysis to the drugs elesclomol (**A**), PF.4708671 (**B**), BIBW2992 (**C**), vorinostat (**D**), camptothecin (**E**), AZD8055 (**F**), SB590885 (**G**), S. trityl.L.cysteine (**H**), BAY61.3606 (**I**), lenalidomide (**J**), MS275 (**k**), and BI. D1870 (**L**), EHT.1864 (**M**), thapsigargin (**N**), AICAR (**O**), SL.0101.1 (**P**), KU.55933 (**Q**), AZD.2281 (**R**), sorafenib (**S**), and A.443654 (**T**) in patients with different OS hypoxia subtypes based on the GDSC database (P value ranking by different drugs with small to large top 20). OS, osteosarcoma; GDSC, Genomics of Drug Sensitivity in Cancer.

Table S1. Hypoxia-related genes list.

Hypoxia related genes					
HIF1A	MDM2	BAD	RUVBL1	ZNF416	RDH13
EGLN1	LDHA	MET	GYS1	NUAK1	CRYBB2
HIF3A	ENO2	IGF2	HDAC4	DLGAP5	UBL4A
EGLN3	TEK	ATR	NUP107	PA2G4	CDC25C
HIF1AN	ARNT2	RELA	MCM6	DHX9	CENPA
EGLN2	LOXL2	VEGFD	CHAF1B	SETMAR	STX10
VEGFA	PRKAA2	BCL2L1	TBC1D7	PKP4	GINS3
EPAS1	RHOA	BIRC5	TCHP	HMBS	NDC80
VHL	PKM	PDK3	FHL3	RPUSD1	PRR11
HYOU1	CYB5A	STC1	CPSF6	ASNS	PRIM1
HILPDA	SPP1	S100B	P4HA2	DDX41	CHRNA5
ARNT	PLK3	FOS	CA12	PSME3	POLA2
SETD2	BHLHE40	HMOX2	BIRC7	DCK	EXOSC8
HIGD1A	PRKAA1	KDM3A	PPFIA4	PFAS	CEP55
EPO	ERO1A	RGS5	FTSJ3	ADCK2	SLC25A19
HIGD2A	WDR83	FURIN	RCN2	CAD	HMMR
CA9	MAPK8	PIK3CA	MRPL24	MSH2	FOXRED2
HIGD1B	ADA	SNRPB	RRAS	MTG1	ZSCAN16
HIGD1C	NAA10	APLN	PHLDA2	TARBP1	INTS3
HIGD2B	PPARG	PLAU	ARV1	DNAJC9	AURKB
TP53	IGFBP1	PARP1	ITGAE	MAD2L1	NUP85
EP300	TSPAN12	ERBB2	DEPDC1B	MELK	CSTF2
CASP3	ZNF408	VIM	BCS1L	ACLY	POLR2L
SLC2A1	ELOB	MAP2K1	NAE1	NXT2	PHF19
MTOR	HP1BP3	AQP1	TIGD5	TAF4	CHCHD3
P4HTM	ENO1	ACE2	RRM2	BUB1B	CDC123
BNIP3	SOD2	ANG	LSM4	FOXD1	NFXL1
CREB1	MAPK3	WTIP	SLC46A1	ZNF114	CDK5
CTNNB1	BECN1	PLOD2	TAP2	HLA-G	SQLE
COL1A1	ADAM17	CCND1	WDR92	MCM7	ANAPC4
CASP9	FLCN	ING4	CATSPER1	HSPH1	NCAPD2
NOS1	FZD4	HSP90B1	RBM45	CENPM	TOP2A
PDK1	ZFP36	GAPDH	TSPAN8	EXOSC2	HABP4
IL6	CDH1	HSPA5	NBEAL2	FERMT1	DNAJB11
TNF	CCN2	EIF2S1	DKK1	EPDR1	FASN
DDIT4	IL10	VCAM1	AHSA1	PRICKLE4	NUPL2
CITED2	TWIST1	NDNF	RANGAP1	CDCA2	FANCI

HMOX1	AHR	PLEKHN1	LRP8	NHP2	HCFC1
IL1B	ICAM1	NRP1	XRCC3	NEIL3	POLE2
CREBBP	IFNG	TRIB3	CENPB	USP1	E2F2
MAPK1	CAT	CDKN1B	RRP7A	GINS2	KIAA0100
CXCL8	CHRDL1	CCL5	MED10	SLC6A8	WDR54
EDN1	NOX4	TERT	PLAC8	ITGA5	NOL11
BDNF	SLC16A1	MUC1	TYMS	CDC25A	FAM13A
BCL2	PGK1	GDF15	NEDD8	ESCO2	NUP62
JUN	IGFBP3	NPPA	PUSL1	SDF2L1	TROAP
NOS2	ETS1	EIF4E	NME1	PDSS1	NUP155
TIGAR	MIF	MANF	ZMYM1	ACTR5	RPIA
PTGS2	ESR1	TIMP1	SLC38A6	NUF2	HSPA14
CYB5R3	SIRT2	TPI1	POLR3K	HSPB1	HSPA1B
AKT1	NGB	PGAM1	RBM15B	SLC7A6	PNCK
TGFB1	VEGFB	PFKP	PRC1	TUFT1	BLM
HIPK2	ANGPT1	PNP	NUP37	CHAF1A	COPS6
MYC	USP28	PSRC1	ACYP1	ALKBH2	ARID3B
NOS3	PLAUR	SEC61G	KIF23	MCM4	MRTO4
REST	LIMD1	AK4	MRPS23	ZNF518A	STRBP
CUL2	CYCS	P4HA1	ZNF232	ZWILCH	GALR2
VEGFC	DICER1	ACOT7	POLQ	PITX1	DTL
RORA	RAC1	GPI	DARS2	ATRIP	RACGAP1
PGF	SLC2A3	TUBB6	CENPF	GTF3C2	CSE1L
CXCR4	CKB	TUBA1C	CENPE	TFCP2	IRX3
KDR	NOL3	CDKN3	SLC2A14	METTL13	PPIH
NDRG1	F3	CTSV	CKS2	CDCA4	TIPIN
RWDD3	HSPA4	TUBA1A	UHRF1	SPAG4	PAQR4
ADM	AR	LRRC42	WDR12	DONSON	STXBP5
CAMK4	DPP4	CHCHD2	DHDH	NEK2	KIF2C
HSP90AA1	BNIP3L	YKT6	SKP2	FKBP14	CACYBP
FAM162A	ZEB1	MAP7D1	GNPDA1	CHEK1	SLC5A6
EGFR	SIAH1	MRPL15	XPO1	NCOA5	SPC25
ANGPT2	NDN	MRPL13	SKA1	KAT2A	TSEN2
MGARP	LOX	MCTS1	HELLS	TPX2	SAAL1
TH	PDGFB	UTP11	TTK	LYAR	CCNA2
LEP	TFRC	KIF4A	MCRS1	ZNF512B	CENPK
FLT1	CDKN1A	MRGBP	SNRPF	POP1	CDCA3
ANGPTL4	ABCB1	KIF20A	MCM3	DUS1L	CDK6
MMP9	HGF	MRPS17	HNRNPA3	TYSND1	RCOR2
LRP5	SNAI1	ESRP1	DCTPP1	BANF1	RRS1
MMP2	ALB	SHCBP1	CDT1	ABCC4	ALG6

SIAH2	CDH5	SLC25A32	PALLD	FDPS	PCNA
EGR1	SMAD3	CORO1C	CCDC14	NCAPG	TUSC2
NDP	SERPINF1	ANLN	MANEAL	MT1X	CCNF
CCL2	HDAC1	MAD2L2	MCM2	EME1	TACC3
MAPK14	KCNB1	ANKRD37	ECT2	FIBCD1	MRPL52
SERPINE1	RBPJ	FOXM1	UBE2T	NUP188	CCNO
SP1	MB	CSRP2	PHF14	CHST3	PSPH
STAT3	TYMP	CD3EAP	TSEN54	TOR3A	PFDN6
CXCL12	FOXO3	CDC20	CASP2	RAN	CCDC138
SOD1	TXN	PHF21A	HIST2H2BE	ZNF503	WISP2
NFKB1	ADORA2B	G6PD	NOP56	SLC7A11	ILF3
EPOR	SLC1A3	RFX5	METTL3	CKAP2	ANGEL2
SIRT1	CD36	PCSK9	NQO1	FBXO5	ABHD12
NFE2L2	ATM	GNB1L	RRP15	GADD45B	DSCC1
SDHB	NFKBIA	ATP5MC1	FAM83D	TMEM45B	TTC13
ALKBH5	SENP1	HASPIN	CDC7	CDCA8	ZNF30
FGF2	UBC	CIP2A	COL7A1	SLBP	MIIP
PTEN	DDIT3	RFC4	CENPO	CA5B	REXO4
INS	HLA-DRB1	CDCA7	FLVCR1	HNRNPA1	EXO1
NOTCH1	FN1	MCM5	FANCG	USP39	DEPDC1
SESN2	PPARA	PPP1R3E	SOX18	PBK	FN3KRP
SFTPC	USP19	CCT5	APOO	RCC2	TTF2
HK2	EIF4EBP1	DNMT1	A4GALT	DNAJB6	SKA2
PIK3CG	UBE2D1	TIMELESS	FAM72D	LBR	SNRPC
ACE	SLC8A1	TCTEX1D2	SLC22A5	PIK3IP1	CENPI
BAX	ATF4	GINS4	CKLF	AURKA	AKR1B10
PSMA7	MMP14	SPIN4	RRM1	PLK1	TLE6
PFKFB4	KLHL20	FANCB	DUT	FEN1	KIF20B
THBS1	HMGB1	FAM111A	CHST10	YEATS2	UCN2
TLR4	RBX1	TMEM201	STXBP6	SMTN	PLIN2
CRP	AJUBA	BRI3BP	ZNF165	SNRPD1	DCLRE1B
ELOC	SART1	WDR4	RFWD3	ZNF142	ASPM
IGF1	ADIPOQ	HSPA1A	DOLK	DTNA	WFS1
XDH	OPRD1	PRR7	POLG2	FAM111B	CCNB1
FUNDC1	BSG	LRP11	TTL	FSCN2	ZMYND19
ENG	TGFB3	TNFRSF21	NCKIPSD	PIR	GMNN
SRC	MBP	RHOQ	RFC3	SAC3D1	

Table S2. ICD and ICP related genes list.

ICD Related Genes		ICP Related Genes			
**ANXA1**	**IFNAR1**	**ADORA2A**	**CD40LG**	**IDO2**	**TNFRSF18**
CALR	IFNAR2	BTLA	CD44	KIR3DL1	TNFRSF25
CXCL10	IFNB1	BTNL2	CD48	LAG3	TNFRSF4
EIF2A	IFNE	CD160	CD70	LAIR1	TNFRSF8
EIF2AK1	IFNW1	CD200	CD80	LGALS9	TNFRSF9
EIF2AK2	LRP1	CD200R1	CD86	NRP1	TNFSF14
EIF2AK3	MET	CD244	CTLA4	PDCD1	TNFSF15
EIF2AK4	P2RX7	CD27	HAVCR2	PDCD1LG2	TNFSF18
FPR1	P2RY2	CD274	HHLA2	TIGIT	TNFSF4
HGF	PANX1	CD276	ICOS	TMIGD2	TNFSF9
HMGB1	TLR3	CD28	ICOSLG	TNFRSF14	VTCN1
IFNA1	TLR4	CD40	IDO1		
IFNA2					

ICD， Immunogenic Cell Death ； ICP， Immune Checkpoint Proteins.

Table S3. Hypoxia related module genes list.

MEyellow		MEturquoise			
**ACE**	**MT1X**	**ACOT7**	**CTSV**	**MAD2L1**	**PRC1**
ANGPT2	NBEAL2	ACYP1	DEPDC1B	MAD2L2	PSRC1
BAX	NFKB1	AURKA	DSCC1	MAP2K1	RACGAP1
BCL2	NOL3	AURKB	DTL	MCM2	RAN
BCL2L1	NOS3	BIRC5	DUT	MCM3	RANGAP1
CCL2	NOX4	BLM	E2F2	MCM5	REXO4
CCL5	NRP1	BRI3BP	EME1	MCM7	RFC3
CCND1	PALLD	BUB1B	EXO1	MCRS1	RFWD3
CDH5	PDGFB	CCNA2	EXOSC2	MRPL13	RRP7A
CITED2	PGF	CCNB1	EXOSC8	MRTO4	SAC3D1
CKLF	PHLDA2	CCNF	FAM72D	NCAPD2	SKA1
CSRP2	PIK3CG	CDC20	FAM83D	NCAPG	SMAD3
CXCL12	PIK3IP1	CDC25A	FANCI	NCKIPSD	SNRPD1
CXCR4	PLAU	CDC25C	FDPS	NEIL3	SNRPF
DPP4	PPARG	CDCA3	FEN1	NEK2	SPC25
EDN1	PRR7	CDCA4	FOXM1	NUP188	TACC3
EIF4EBP1	RGS5	CDCA7	GINS2	NUP37	TIMELESS
ENG	RRAS	CDCA8	GINS3	NUP85	TIPIN
EPAS1	SDF2L1	CDKN3	GINS4	PA2G4	TMEM201

EPDR1	SLC38A6	CDT1	GMNN	PAQR4	TPX2
ETS1	SOX18	CENPA	GTF3C2	PCNA	TROAP
FLT1	SPP1	CENPM	HDAC1	PFAS	TSEN54
FZD4	SRC	CENPO	HMBS	PHF19	TUBA1C
HIF1A	TAP2	CHCHD3	HNRNPA1	PKP4	TYMS
HLA-DRB1	TEK	CHRNA5	KIF20A	PLK1	UBE2T
HMOX1	TLR4	CKS2	KIF23	POLA2	XRCC3
ICAM1	TNF	COPS6	KIF2C	POLQ	ZMYND19
IL10	TSPAN12	CSE1L	LRRC42	PPIH	ZWILCH
IL1B	TYMP				
KDR	WFS1				
MBP	ZEB1				
MIF	ZFP36				
MMP9					

Table S4. GO and KEGG enrichment analysis results of HRMGs.

Ontology	ID	Description	GeneRatio	BgRatio	p.adjust	qvalue
BP	GO:0050900	leukocyte migration	20/170	384/18800	1.72E-08	1.04E-08
BP	GO:0001666	response to hypoxia	19/170	286/18800	1.48E-09	8.99E-10
BP	GO:2001234	negative regulation of apoptotic signaling pathway	17/170	230/18800	2.99E-09	1.81E-09
BP	GO:0060326	cell chemotaxis	17/170	315/18800	1.55E-07	9.4E-08
BP	GO:0050727	regulation of inflammatory response	17/170	394/18800	3.18E-06	1.92E-06
CC	GO:0000793	condensed chromosome	21/174	255/19594	6.76E-13	5.54E-13
CC	GO:0072686	mitotic spindle	13/174	160/19594	6E-08	4.91E-08
CC	GO:0031261	DNA replication preinitiation complex	7/174	12/19594	1.12E-10	9.19E-11
CC	GO:0043596	nuclear replication fork	5/174	28/19594	9.25E-05	7.58E-05
CC	GO:0097431	mitotic spindle pole	4/174	36/19594	0.0034	0.0028
MF	GO:0048018	receptor ligand activity	14/175	489/18410	0.0085	0.0068
MF	GO:0005126	cytokine receptor binding	13/175	272/18410	0.0002	0.0001
MF	GO:0003678	DNA helicase activity	8/175	72/18410	6.01E-05	4.8E-05
MF	GO:0070851	growth factor receptor binding	6/175	139/18410	0.0289	0.0231
MF	GO:0008009	chemokine activity	4/175	49/18410	0.0190	0.0152
KEGG	hsa05166	Human T-cell leukemia virus 1 infection	19/106	222/8164	5.08E-09	2.66E-09
KEGG	hsa04218	Cellular senescence	12/106	156/8164	1.6E-05	8.41E-06
KEGG	hsa04010	MAPK signaling pathway	11/106	294/8164	0.0067	0.0035
KEGG	hsa04657	IL-17 signaling pathway	5/106	94/8164	0.0212	0.0111
KEGG	hsa04659	Th17 cell differentiation	5/106	108/8164	0.0342	0.0179

HRMGs， Hypoxia related module genes ；GO，Gene Ontology；BP，biological process；CC，cellular component；MF，molecular function；KEGG，Kyoto Encyclopedia of Genes and Genomes。

DETAILS OF THE STUDY METHODS

Data extraction and pre-processing

We downloaded the expression profile data of the OS GDC TARGET-OS dataset through the Therapeutically Applicable Research To Generate Effective Treatments (TARGET) database (https://xena.ucsc.edu/), including HTSeq-Counts (n=88) expression profile data and HTSeq-FPKM (n=88) expression profile data. At the same time, we also downloaded the patient clinical data (n=524) and survival data (n=288) of OS. Samples of OS patients with expression profile data, survival data, and clinical data were included in this study, for a total of 84 OS patients, including 63 OS patients without metastasis (group: Unmetastases) and 21 OS patients with metastasis (group: Metastases).

We also downloaded the expression profile datasets GSE21257[14] and GSE14359[15] of OS patients from the Gene Expression Omnibus (GEO) database (https://www.ncbi.nlm.nih.gov/geo/)[16] by using the R package Equerry. The samples in the two datasets were all derived from Homo sapiens. The samples of the GSE21257 dataset were the genome-wide gene expression profiling of OS tumour tissues from prechemotherapy biopsies of OS patients, a total of 53 samples, including 19 samples of OS patients without metastases within 5 years (group: Unmetastases) and 34 samples of OS patients with metastases within 5 years (group: metastases). Table **1** shows the specific dataset information.

We collected HRGs from the GeneCards database (https://www.genecards.org/). The GeneCards database [17] provides comprehensive information on human genes. With the word "hypoxia" as the search keyword and "protein coding" and "relevance score>3" as the screening criteria, we obtained 279 HRGs. We also used "Hypoxia" as the search keyword on the Molecular Signatures Database (MSigDB)[18] website, and 50 HRGs were collected from the reference genes of BUFFA HYPOXIA METAGENE.v2022.1.Hs. In addition, we used "hypoxia" as the search keyword to obtain the list of HRGs in the published literature on the PubMed website [19]. The published literature on the PubMed website contains 397 HRGs in total. After merging and deduplication, we obtained 689 HRGs. See **Table S1** for details.

ICD is a specific variant of regulated cell death driven by stress that can induce adaptive immunity against dead cell antigens. ICP molecules refer to ligand–receptor pairs that exert inhibitory or stimulatory effects on immune responses. Immune checkpoints play an important immunoregulatory role in maintaining immune homeostasis and preventing autoimmunity. We obtained 25 ICD genes and 46 ICP genes from the published literature on the PubMed website [20,21]. The specific gene names are shown in **Table S2**.

Osteosarcoma-related overexpressed genes

To identify the potential mechanism and related biological features and pathways of genes in OS, we first used the limma package [22] to perform differential analysis between samples grouped by Metastases/Unmetastases of the OS dataset TARGET-OS, and then we apply a linear model to the OS dataset TARGET-OS, GSE21257, GSE14359 to identify the overexpressed genes between the Metastases and the Unmetastases. Finally, we performed differentially analyse the expression profile data of the TARGET-OS, GSE21257, and GSE14359 datasets, and the genes screened out with the criteria of $|logFC|>0.5$ and p value < 0.05 were overexpressed genes.

The resulting overexpressed genes in the TARGET-OS, GSE21257, and GSE14359 datasets were intersected, and a Venn diagram was drawn. The genes are obtained from a minimum of two sets of data and are identified as overexpressed genes in OS. The OS dataset differential analysis results were exhibited by volcano plots plotted by the R package ggplot2.

Prognostic survival analysis of overexpressed genes and antigen-presenting cell correlation analysis

We combined clinical prognostic information of TARGET-OS dataset samples to perform an analysis of the relationship between the overexpressed genes in the TARGET-OS and GSE21257 datasets and the prognostic outcome of OS patient survival and plotted the prognostic survival Kaplan–Meier (KM) curve. Overexpressed genes with a prognostic survival KM curve p value < 0.05 and the same trend were then included in the subsequent study.

Immune infiltration analysis was performed on the TARGET-OS OS tissue FPKM format dataset matrix using the ssGSEA algorithm in the GSEA package [23] to evaluate the infiltration abundance of 28 immune cells in the tumor microenvironment. Then, the correlation of potential tumor antigens (overexpressed genes) with major antigen-presenting cells such as dendritic cells, macrophages, B cells, etc. was calculated by Spearman correlation analysis.

Identification and validation of hypoxia subtypes

A univariate Cox regression analysis was first performed based on the expression of HRGs in the TARGET-OS dataset, and the results were filtered at p value < 0.05 for inclusion in subsequent consensus clustering analysis. Consensus clustering [24] is a resampling-based algorithm to identify each sample and its subgroup numbers and verify the plausibility of clustering. We used the consensus clustering method of the ConsensusClusterPlus package [25] to analyse the HRGs after screening in the TARGET-OS dataset, identifying different OS hypoxia subtypes. The clustering of samples from the different OS hypoxia subtypes in the TARGET-OS dataset was quantified by principal component analysis (PCA), and the analysis results are presented by PCA plots. KM curves were then plotted to demonstrate the correlation between the OS hypoxic subtype and patient prog-

nosis. The same approach was taken to analyse the GSE21257 dataset to validate the prognostic diagnostic effect of the OS hypoxia subtype of the TARGET-OS dataset.

Differential analysis of ICD and ICP expression in different hypoxia subtypes and immune evaluation

Tumor microenvironment cells as well as the degree of infiltration of immune cells and stromal cells in the tumor have a significant impact on prognosis. The ESTIMATE package leverages the unique properties of transcriptional profiles of cancer samples to infer the contents of tumor cells as well as different infiltrating normal cells. To better understand the prognostic impact of genes involved in immune and stromal cells, the immune activity of the TARGET-OS and GSE21257 datasets was evaluated based on the ESTIMATE package. The immune score for each sample in the OS dataset was obtained based on the gene expression profile quantitative analysis of immune activity (immune infiltration level) in OS patient samples. The differences in immune infiltration characteristics of OS patient samples between different OS hypoxia subtypes from the TARGET-OS and GSE21257 datasets were compared.

We performed a differential analysis of the expression of ICD and ICP genes in OS patient samples between different OS hypoxia subtypes in the TARGET-OS and GSE21257 datasets. The group differences in ICD and ICP genes among different OS hypoxia subtypes were compared by Mann–Whitney U test (Wilcoxon rank sum test) calculations. The analysis results were exhibited by boxplots, and the results were all considered significantly different at a p value < 0.05.

Identification of immune infiltrating cells among different OS hypoxia subtypes in OS datasets

The ssGSEA algorithm was used to quantify the relative abundance of each immune cell infiltrate. The enrichment score calculated from ssGSEA was utilized to represent the relative abundance of immune cell infiltration in each sample [26,27]. The enrichment score calculated by ssGSEA algorithm analysis in the GSEA package was used to represent the infiltration level of different types of immune cells in each sample. The differences in the infiltration abundance of immune cells between the different OS hypoxia subtypes of OS datasets (TARGET-OS and GSE21257 datasets) were exhibited by heatmaps.

Hypoxia landscape construction of the tumor microenvironment (pseudotime analysis)

Pseudotime analysis, according to the time-specific change in the amount of gene expression of different cell subpopulations with virtual out, constructs cell lineage development to determine the order and trajectory of transformation and succession between cells; that is, pseudotime analysis remodels the changing course of cells over time by constructing the trajectory of change among cells. We performed dimension reduction analysis based on the inverse graph embedding (DDRTree) algorithm, utilizing the dimension reduction function of the Monocle package (version 2.0) [28] with Gaussian distribution, to visualize the distribution in individual patients of the OS hypoxic subtype of the TARGER-OS dataset. The maximum number of components was set to five, and the discriminant dimension reduction method with the inverse graph embedding (DDRTree) algorithm was used for dimension reduction analysis. Finally, color-coded functional maps of hypoxic subtypes of OS cell trajectories were used to visualize the hypoxic landscape.

Weighted gene coexpression network analysis (WGCNA) of different OS hypoxia subtypes in the TARGET-OS dataset

WGCNA uses the normalized expression correlation coefficient of each gene to evaluate the coexpression relationship between each gene and defines the gene with a coexpression relationship as a module. Through this way of thinking, complex high-throughput data can be converted into simple modules, and dimensionality reduction processing is carried out to a certain extent. Finally, the relationship between these gene coexpression modules and clinical phenotype can be found, and then the biological significance of this module can be found [29]. We used the WGCNA package [30] to analyse the expression matrix of anoxic-related genes in the TARGET-OS dataset. RsquaredCut is set to 0.85 to obtain the best soft threshold. The minimum number of module genes is set to 20, and the module merges shear height is set to 0. In this way, the coexpression modules related to different OS hypoxia subtype sample genes were obtained. We will conduct subsequent analysis on the module genes contained in modules with correlation $|COR| \geq 0.3$ and p value<0.05 in the module analysis results.

Gene functional enrichment analysis and pathway enrichment analysis

Gene Ontology (GO) [31] analysis is a common method to perform large-scale functional enrichment studies, including biological process (BP), molecular function (MF), and cellular component (CC). The Kyoto Encyclopedia of Genes and Genomes (KEGG) [32] is a widely used database that stores information about genomes, biological pathways, diseases, and drugs. GO annotation analysis of module genes in the OS hypoxia subtype of the TARGET-OS dataset was performed by the R package clusterProfiler with input filtering criteria p.adj < 0.05 and FDR value (q.value) < 0.5 as statistically significant and Benjamini–Hochberg as a correction method for p value.

Drug sensitivity analysis

The change in the cancer genome strongly affects the clinical response to treatment and, in many cases, is an effective biomarker of drug treatment response. The Genomics of Drug Sensitivity in Cancer (GDSC) [33] database (www.cancerRxgene.org) is the largest public resource for information on drug sensitivity and drug response molecular markers of cancer cells. The

cancer drug sensitivity genomics database can be used to find tumor drug response data and sensitive markers of the genome. IC50 values were calculated by the prophetic [34] algorithm to predict the sensitivity of OS patients with different OS hypoxia subtypes in the TARGET-OS dataset to common anticancer drugs or small molecule compounds.

Clinical sample validation

Quantitative real-time PCR will be used to evaluate the expression of four tumor antigens, and immunohistochemistry will be performed on normal bone tissue and osteosarcoma tissue to further verify our analysis. Transient transfection was used to interfere with four tumor antigen genes, and the interference effect was verified by the Western blot experiment. Cell counting kit-8 assay and Transwell assays were used to evaluate the proliferation and invasion ability of cells before and after interference, respectively.

Statistical analysis

The pseudotime analysis of this article is based on the R software (Version 4.1.1) Monocle2.0 package. All other data processing and analyses were based on R software (Version 4.2.2). Continuous variables are displayed as the mean ± standard deviation. The Wilcoxon rank sum test was used for comparisons between two groups; the Kruskal–Wallis test was used for comparisons of three groups and above. If not specified, the correlation coefficients between different molecules were calculated by Spearman correlation analysis, and all statistical P values were bilateral, with $P<0.05$ as statistically significant.

Some core algorithm formulas of this study.

Identification of osteosarcoma-associated overexpressed genes used the limma package in R.

Immune infiltration analysis utilizes the ssGSEA algorithm in the GSEA package.

Consensus clustering used the consensus clustering method of the ConsensusClusterPlus package. Consensus clustering method: Let G = (V, E) be a graph where *V* denotes the set of nodes and *E* the set of edges of *G*. Let C = (C1,..., Ck) be a consensus clustering with *K* clusters and |Ci| the number of nodes classifed in cluster Ci. Let us also note *w*(*e*) the weight of a given edge, W(eii) the sum of weights of the edges internal to Ci cluster (connecting vertices from Ci), W(eij) the sum of weights between clusters Ci and Cj (connecting a vertex from Ci to a vertex from Cj) and max(wIG) the maximum possible weight on the edges of the given integration graph (the maximum possible number of supports, also corresponding to the number of input clusterings being fused). We therefore defne the *wMQ* index computed for a consensus clustering *C* obtained on the integration graph *IG* as:

$$vMQ(IG,C) = \frac{1}{k \times max(w_{IG})}\sum_{i=1}^{k}\left(\frac{2\times W(e_{ii})}{|C_i|\times(|C_i|-1)} - \frac{1}{k-1}\sum_{j\neq i}\frac{W(e_{ij})}{|C_i|\times|C_j|}\right)$$

Pseudotime analysis, utilizing the dimension reduction function of the Monocle package (version 2.0), and the discriminant dimension reduction method with the inverse graph embedding (DDRTree) algorithm was used for dimension reduction analysis.

Weighted gene coexpression network analysis: In unweighted networks, *ClusterCoefi* equals 1 if and only if all neighbors of gene *i* are also linked to each other. For weighted networks, $0 \leq aij \leq 1$ implies that $0 \leq ClusterCoefi \leq 1$. The mean clustering coefficient has been used to measure the extent of module structure present in a network.

$$\text{ClusterCoef}_{\text{i}} = \frac{\sum_{l\neq i}\sum_{m\neq i,} l^a\ il^a lm^a mi}{\{(\sum_{l\neq i} a_{il})^2 - \sum_{l\neq i}(a_{il})^2\}}.$$
